# Benefits of biological nitrification inhibition of *Leymus chinensis* under alkaline stress: the regulatory function of ammonium-N exceeds its nutritional function

**DOI:** 10.3389/fpls.2023.1145830

**Published:** 2023-05-15

**Authors:** Gui Wang, Lihui Zhang, Zihan Guo, Dongfang Shi, Huiliang Zhai, Yuan Yao, Tianxue Yang, Shuquan Xin, Haiying Cui, Junqin Li, Jianying Ma, Wei Sun

**Affiliations:** ^1^ Institute of Grassland Science, Key Laboratory of Vegetation Ecology of the Ministry of Education, Jilin Songnen Grassland Ecosystem National Observation and Research Station, Northeast Normal University, Changchun, China; ^2^ School of Life Sciences, Changchun Normal University, Changchun, Jilin, China; ^3^ Analysis and Testing Center, Changchun Normal University, Changchun, Jilin, China; ^4^ Key Laboratory of Geographical Processes and Ecological Security in Changbai Mountains, Ministry of Education, School of Geographical Sciences, Northeast Normal University, Changchun, China

**Keywords:** ammonium, nitrate, nutrition, biological nitrification inhibition, adaptation to alkaline stress

## Abstract

**Introduction:**

The production of root exudates with biological nitrification inhibition (BNI) effects is a strategy adopted by ammonium-N ( $\text{N}{{\text{H}}_{4}}^{+}‐\text{N}$ ) tolerant plant species that occur in N-limited environments. Most knowledge on BNI comes from plant species that occur in acidic soils.

**Methods:**

Here, combining field sampling and laboratory culture, we assessed the BNI-capacity of *Leymus chinensis*, a dominant grass species in alkaline grasslands in eastern Asia, and explored why *L. chinensis* has BNI ability.

**Results and discussion:**

The results showed that *L. chinensis* has strong BNI-capacity. At a concentration of 1 mg mL^-1^, *L. chinensis*’ root exudates inhibited nitrification in soils influenced by *Puccinellia tenuiflora* by 72.44%, while DCD only inhibited it by 68.29%. The nitrification potential of the soil of *L. chinensis* community was only 53% of the *P. tenuiflora* or 41% of the *Suaeda salsa* community. We also showed that the supply of $\text{N}{{\text{H}}_{4}}^{+}‐\text{N}$ driven by *L. chinensis*’ BNI can meet its requirements . In addition, $\text{N}{{\text{H}}_{4}}^{+}‐\text{N}$ can enhance plant adaptation to alkaline stress by regulating pH, and in turn, the uptake of nitrate-N ( ${{\text{NO}}_{3}}^{‐}‐\text{N}$ ). We further demonstrated that the regulatory function of $\text{N}{{\text{H}}_{4}}^{+}‐\text{N}$ is greater than its nutritional function in alkaline environment. The results offer novel insights into how *L. chinensis* adapts to high pH and nutrient deficiency stress by secreting BNIs, and reveal, for the first time, differences in the functional roles of $\text{N}{{\text{H}}_{4}}^{+}‐\text{N}$ and ${{\text{NO}}_{3}}^{‐}‐\text{N}$ in growth and adaptation under alkaline conditions in a grass species.

## 1 Introduction

Soil pH regulates soil nutrient storage and supply, some micronutrients can become more available and more toxic to plants in acidic soil, and phosphorus and most micronutrients become less available in alkaline soil, thus influences plant productivity in terrestrial ecosystems (Slessarev et al., 2016; Weil and Brady, 2017). Soil pH can play a role in nitrogen (N) availability through affecting N forms and N cycling processes (Neina, 2019). Despite the fact that plants can uptake organic-N, ammonium-N ( $\text{N}{{\text{H}}_{4}}^{+}‐\text{N}$ ) and nitrate-N ( ${{\text{NO}}_{3}}^{‐}‐\text{N}$ ) are the primary N forms utilized by plants (Subbarao et al., 2012). However, approximately 95% of total soil N is in forms of soil organic nitrogen (SON) (Schulten and Schnitzer, 1998), and the vast majority of SON must be transformed into inorganic-N (IN) through mineralization before it can be utilized by plants. In addition, high pH inhibits ammonification (Pathak and Rao, 1998) but enhances nitrification (Ste-Marieab and Paré, 1999; Kyveryga et al., 2004), which potentially leads to N loss through leaching or denitrification. Nitrification converts the relatively static $\text{N}{{\text{H}}_{4}}^{+}‐\text{N}$ form into the highly mobile ${{\text{NO}}_{3}}^{‐}‐\text{N}$ form in non-alkaline soil, which may be lost easily via leaching or transformed into a gaseous state through denitrification; such processes not only lead to low N utilization efficiency (NUE) in agropastoral systems but also environmental problems such as water pollution and nitrous oxide (greenhouse gas) emissions (Subbarao et al., 2006b; Coskun et al., 2017). Moreover, ammonia (NH_3_) volatilization is more pronounced at high pH levels (Weil and Brady, 2017). Therefore, alkaline soil associated high pH reduces N bioavailability.

Some dominant plant species in natural climax ecosystems, such as *Brachiaria humidicola*, have been reported to produce organic compounds that inhibit nitrifier activity in soil (Subbarao et al., 2012), which has been termed biological nitrification inhibition (BNI) since the early 1900s (Nardi et al., 2020). BNI is considered as a promising strategy for application in the improvement of the eco-efficiency of agropastoral systems and climate change mitigation by reducing agronomic N losses (Moreta et al., 2014; Otaka et al., 2021). Extensive studies have been carried out on biological nitrification inhibitors (BNIs) derived from plant root exudates (Subbarao et al., 2006a). For example, it has been reported that *Hyparrhenia diplandra* (Lata et al., 2004; Srikanthasamy et al., 2022), *Sorghum bicolor* (Zakir et al., 2008; Subbarao et al., 2013; Bozal-Leorri et al., 2023), *B. humidicola* (Gopalakrishnan et al., 2007; Subbarao et al., 2007a; Subbarao et al., 2009; Nakamura et al., 2020), *Oryza sativa* (Sun et al., 2016; Lu et al., 2019; Zhang et al., 2019), *Zea mays* L. cv Honey bantam (Otaka et al., 2021) and *Leymus racemosus* (Subbarao et al., 2007b) can inhibit soil nitrification via BNIs in root exudates. To date, most studies have focused on the identification and synthesis of specific BNIs, unraveling their mechanisms of action, and exploring how they could be used in soil remediation (Subbarao et al., 2012; Subbarao et al., 2013; Coskun et al., 2017).

Several studies with synthetic nitrification inhibitors (SNIs) on crops have demonstrated that SNIs suppress nitrification, improve N recovery, and increase economic yields significantly, while BNIs has the potential to revolutionize the efficiency of N uptake and utilization and minimize N losses (Subbarao et al., 2006b). It was suggested that the suppression of nitrification and the maintenance of N fertilizer in its reduced form are critical steps to increase fertilizer-N retention in soils and to improve the NUE of crops (Sun et al., 2016). However, these studies were conducted on plants growing in environments with acidic soils such as tropical savannas or cropland (Subbarao et al., 2009; Subbarao et al., 2012; Sun et al., 2016). Not much information is available on whether plants have BNI in an alkaline environment, especially in ecosystems limited by N, such as the *L. chinensis* meadow steppe (Bai et al., 2010). Thus, assessing the BNI capacity of *L. chinensis* and elucidating the mechanisms of the adaptation of *L. chinensis* to alkali stress through BNI will facilitate the restoration of the degraded saline-alkaline meadow steppe.

*L. chinensis* is widely distributed in eastern region of the Eurasian steppe (Zhang et al., 2014), where soils are often experiencing alkalinization. The species is highly tolerant of drought, low soil fertility, and high pH, and it often forms monodominant stands (Wang et al., 2004). It has been reported that *L. chinensis* is often limited by low soil N availability and prefers $\text{N}{{\text{H}}_{4}}^{+}‐\text{N}$ over ${{\text{NO}}_{3}}^{‐}‐\text{N}$ for its growth (Li et al., 2018). However, soils with high pH are prone to nitrification (Kyveryga et al., 2004; Sahrawat, 2008), which is not conducive to the preservation of $\text{N}{{\text{H}}_{4}}^{+}‐\text{N}$ because of nitrification and NH_3_ volatilization (Mills et al., 1974). Consequently, there is a paradox for *L. chinensis* inhabiting alkaline environments, since the species has to reconcile its preference for $\text{N}{{\text{H}}_{4}}^{+}‐\text{N}$ (Li et al., 2018) with inhabiting environments that are not conducive to the existence of $\text{N}{{\text{H}}_{4}}^{+}‐\text{N}$ . To date, it remains unclear how *L. chinensis* addresses this incongruity.

A previous study reported that *L. racemosus*, a relative of *L. chinensis*, exhibits high BNI capacity (Subbarao et al., 2007b); if *L. chinensis* also has high BNI capacity, it could be the solution to the aforementioned paradox. Therefore, we hypothesized that *L. chinensis* has BNI-capacity. Previous studies have revealed that plant responses to ${{\text{NO}}_{3}}^{‐}‐\text{N}$ can be affected by the co-provision of $\text{N}{{\text{H}}_{4}}^{+}‐\text{N}$ , and $\text{N}{{\text{H}}_{4}}^{+}‐\text{N}$ responses are altered by ${{\text{NO}}_{3}}^{‐}‐\text{N}$ ; the interactions between ${{\text{NO}}_{3}}^{‐}‐\text{N}$ and $\text{N}{{\text{H}}_{4}}^{+}‐\text{N}$ are likely to optimize N utilization (Li et al., 2013; Hachiya and Sakakibara, 2017). Therefore, we hypothesized that $\text{N}{{\text{H}}_{4}}^{+}‐\text{N}$ uptake facilitates the uptake of ${{\text{NO}}_{3}}^{‐}‐\text{N}$ by *L. chinensis*, and improve the productivity of *L. chinensis*. We proposed that *L. chinensis* needs $\text{N}{{\text{H}}_{4}}^{+}‐\text{N}$ through the BNI, then uses the H^+^ produced from $\text{N}{{\text{H}}_{4}}^{+}‐\text{N}$ metabolism to reduce the pH of the rhizosphere, and promote the uptake of ${{\text{NO}}_{3}}^{‐}‐\text{N}$ via the establishment of the proton motive force. via, To test our hypotheses, 4 experiments were carried out: (1) a field experiment on differences in soil nitrification ability along a vegetation succession stage; (2) an experiment on the assessment of the nitrification inhibition ability of *L. chinensis* root exudates; (3) an experiment to assess the rhizosphere effects of *L. chinensis* on soil nitrification; and (4) a hydroponic experiment to test the functions of $\text{N}{{\text{H}}_{4}}^{+}‐\text{N}$ and ${{\text{NO}}_{3}}^{‐}‐\text{N}$ in *L. chinensis* growth and adaptation to alkali stress.

## 2 Materials and methods

### 2.1 Differences in soil nitrification along a vegetation succession stage

We used space-for-time substitution method (Blois et al., 2013) to investigate the effects of vegetation succession on soil N availability and nitrification intensity in a natural saline-alkali meadow steppe in northeast China.

#### 2.1.1 Study site and soil sampling

**Figure 1:**
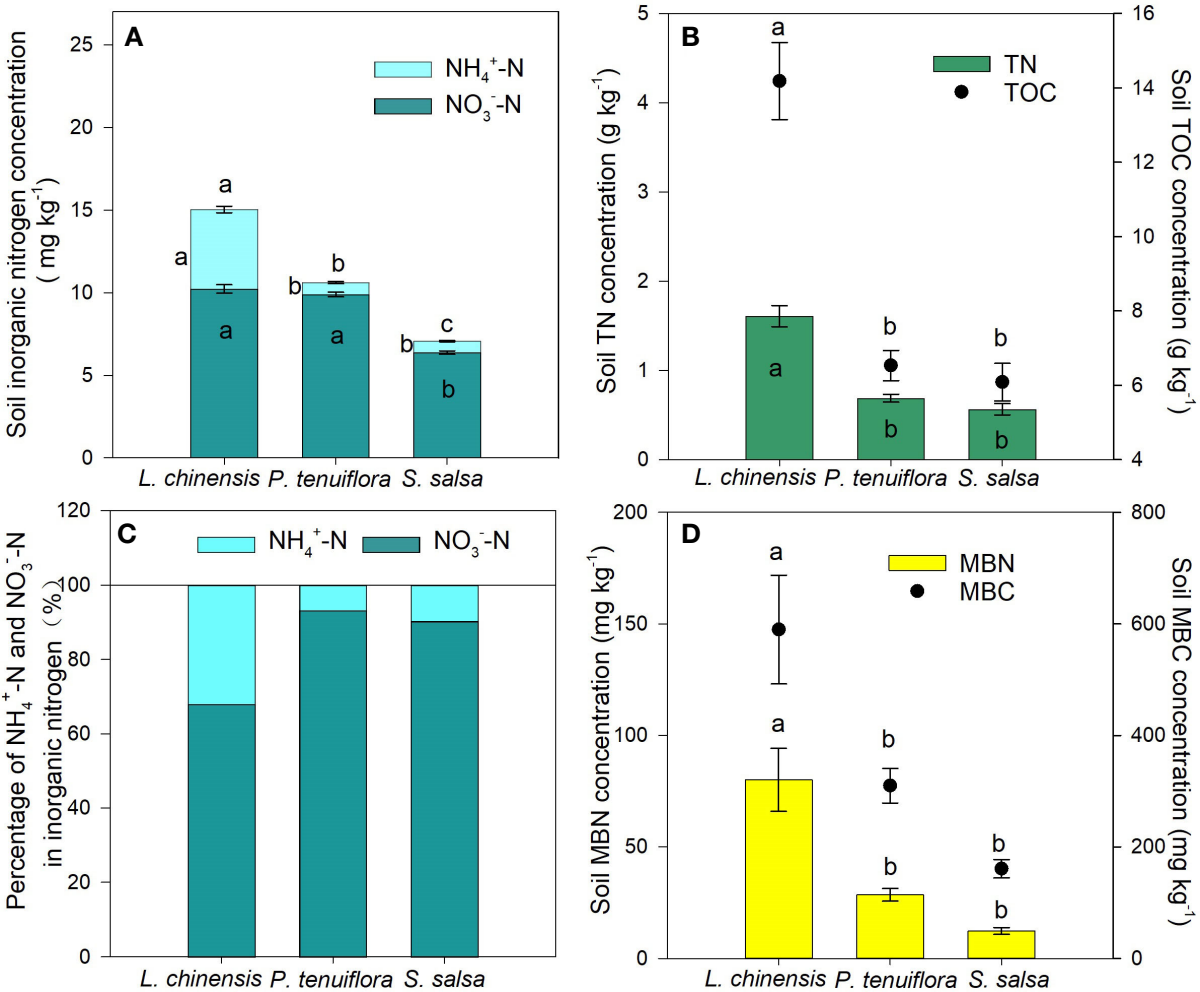
Differences in the concentrations of inorganic nitrogen **(A)**, soil total nitrogen (TN) and soil total organic carbon **(B)**, percentage of $\text{N}{{\text{H}}_{4}}^{+}‐\text{N}$ and ${{\text{NO}}_{3}}^{‐}‐\text{N}$ in inorganic nitrogen **(C)**, and soil microbial biomass nitrogen (MBN) and soil microbial biomass carbon (MBC) **(D)** of 0-10 cm soil among the three plant communities (*L. chinensis*: the final stage of vegetation succession; *P. tenuiflora:* the middle stage of succession; *S. salsa*: the initial stage of succession). Data are means ± 1 SE (n = 5). Different letters denote significant differences among the succession stages (*P* < 0.05).

The study area is located in the west of Songliao Plain and the eastern margin of Horqin Sandy Land (43°79′ N and 123°69′ E; Dongtai pasture of Zuoling Village, Wohu Town, Shuangliao City, Jilin Province, China), which is characterized by a temperate continental, semiarid monsoonal climate with a mean annual temperature and total precipitation of 6.5°C and 395.5 mm, respectively; more than 75% of the precipitation and accumulated temperature are concentrated in the period from May to September (1968 - 2019); and the soil type is characterized as Chernozem with high sodic and saline contents, according to the Food and Agriculture Organization (FAO) classification (Yao et al., 2022). It is a typical saline-alkali meadow steppe with an elevation of 150 m and soil pH ranging from 8 to 10. Details of the soil properties and vegetation features of the sampling sites are shown in **Table S1** and **Figure 1**. We selected three vegetation types at different stages of succession, which was driven primarily by soil pH and soil electrical conductivity (EC), including the *Suaeda salsa* community at the initial stage of succession, the *Puccinellia tenuiflora* community in the middle stage of succession, and the *L. chinensis* community (climax community for the studied area). For each plant community, there were five plots, and each plot had an area of 5 m × 5 m.

For each plant community, soils (0 - 10 cm) were sampled at five locations using a soil core (5 cm in diameter) and mixed into a composite sample in July 2019. To ensure the representativeness of soil sampling, we centered soil cores on the roots of each dominant plant of the *S. salsa*, the *P*. *tenuiflora*, or the *L. chinensis*. The composite soil samples were sieved (2 mm) and visible roots were removed. Each composite soil sample was divided into three parts: one part was stored at 4°C and subsequently used for nitrification potential determination; another part was stored at -20°C for nutrient analysis; the last part was air-dried for measurement of pH and total nitrogen.

#### 2.1.2 Determination of the properties of soil

The concentration of $\text{N}{{\text{H}}_{4}}^{+}‐\text{N}$ was determined using the modified indophenol blue spectrophotometry method (Gao et al., 2013). In brief, the frozen- stored soil samples (equivalent to 1 ± 0.1 g dry weight) were weighed into a 10 mL centrifuge tube. After the addition of 5 mL of 2 M KCl solution, the tube was sealed and extracted for 30 min through oscillating up and down at room temperature, then centrifuged at 9569 g for 10 min at 4°C. An aliquot of supernatant (50 μL) was pipetted into a microwell of the 96-well plate, and diluted with 150 μL ultrapure water. Subsequently, 25 μL of 1% phenol solution (containing 1% sodium nitroferricyanide as catalyst) and 25 μL of 0.3% sodium dichloroisocyanurate dihydrate solution (in 2% NaOH) were added, and the mixture was incubated at 35°C for 30 min. The absorbance values of the wells were then read with a microplate reader (Synergy HT, BioTek, Winooski, VT, USA) at a wavelength of 625 nm.

The concentration of ${{\text{NO}}_{3}}^{‐}‐\text{N}$ was determined using the modified ultraviolet spectrophotometry method (Edwards et al., 2001). The extraction procedures were similar to the measurement of soil $\text{N}{{\text{H}}_{4}}^{+}‐\text{N}$ concentration but replaced a 2 M KCl solution with a 0.01 M CaCl_2_. An aliquot of supernatant (2 mL) was transferred into a 1 cm path length quartz cuvette, and 80 μL of 10% H_2_SO_4_ was added to eliminate interference from ions of OH^-^, $\text{C}{{\text{O}}_{3}}^{2‐}$ and $\text{HC}{{\text{O}}_{3}}^{‐}$ . The absorbance of the supernatant was measured with a UV spectrometer (T6, Beijing General Instrument Co., Ltd, Beijing, China) at wavelengths of 210 nm (A_210_) and 275 nm (A_275_). Soil total inorganic N (TIN) was calculated as the sum of the concentrations of ${{\text{NO}}_{3}}^{‐}‐\text{N}$ and $\text{N}{{\text{H}}_{4}}^{+}‐\text{N}$ .

Soil total nitrogen (TN) concentration was determined by the Kjeldhal method. In brief, soil samples (approximately 0.5 g) were weighed into the digestion tube. After the addition of 1.8 g catalyst (K_2_SO_4_: CuSO_4_·5H_2_O: Selenium powder = 10: 1: 0.1) and 5 mL of concentrated H_2_SO_4_, the digestion tubes were heated at 150°C for 30 min, and followed by heating at 400°C for 150 min. Soil TN concentration was measured using a Foss automated Kjeltec™ instrument (Kjeltec™ 8400, FOSS, Hillerød, Denmark).

Soil total organic carbon (TOC) was determined by an element analyser (Vario TOC, Elementar, Hanau, Germany), after treatment with 2 M HCl to remove inorganic carbon.

Soil microbial biomass carbon (MBC) and nitrogen (MBN) were determined using the soil subsamples stored at -20°C with the chloroform fumigation-extraction method. After chloroform fumigation, the fumigated and un-fumigated soils were extracted with 0.5 M K_2_SO_4_ (soil: deionized water = 1: 10, w: v), soil MBC and MBN (the differences between the fumigated and un-fumigated soil samples) were determined with an element analyser (Vario TOC, Elementar, Hanau, Germany). The corrective coefficient for MBC and MBN was 0.38 (Joergensen, 1996) and 0.54 (Brookes et al., 1985), respectively.

The modified shaken soil-slurry method (Hart et al., 1994) was used to determine nitrification potential. Briefly, fresh soil (equivalent to 3.0 g dry weight) was transferred into Erlenmeyer flasks and filled with 30 mL phosphorous-nitrogen (PN) solution. The PN solution had final concentrations of 1/15 M PO_4_^3-^ (v 1/15 M Na_2_HPO_4_: v 1/15 M KH_2_PO_4_ = 9.5: 0.5) and 75 mM $\text{N}{{\text{H}}_{4}}^{+}‐\text{N}$ at pH 8.04. The soil-slurries were shaken for 30 min on a shaker at 180 rpm at 28°C. Afterward, 1 mL aliquots were taken and stored at -37°C as 0 h samples, and then the remaining soil-slurries were incubated under continuous shaking at 28°C and 1 mL aliquots were taken every 24 h until 72 h, and stored at -37°C. Subsequently, the samples were thawed and centrifuged at 9569 g for 10 min at 4°C. Nitrite-N ( ${{\text{NO}}_{2}}^{‐}‐\text{N}$ ) concentration in the soil slurry was determined using a colorimetric microplate assay; the nitrification potential was expressed by changes in ${{\text{NO}}_{2}}^{‐}‐\text{N}$ concentration of per mL soil-slurry for a given period of time (O’Sullivan et al., 2017).

### 2.2 Nitrification inhibition experiment of root exudates of *L. chinensis*

We collected root exudates of *L. chinensis* and used the shaken soil-slurry method (Hart et al., 1994) to test the nitrification inhibition capacity of the root exudates.

#### 2.2.1 Root exudate collection

A device (**Figure S1**) was designed for collecting root exudates of *L. chinensis* using the double layer culturation method, with the upper layer filled with soil used to grow plants on a nylon mesh; the soil was collected from the plots of the *L. chinensis* community, and the lower layer filled with water. 7 clonal propagules of *L. chinensis* were transplanted into the upper soil layer, and then maintained at open air conditions during a complete growing season from April to July, watering the soil every other day with weighing method to make the soil water content reach 80% of the field water-holding capacity (WHC). Root exudates were collected after 4 months of growth. To achieve this, the lower layer was replaced with fresh water for 24 hours once an adequate amount of plant roots had grown throughout the upper soil layer into the lower layer. . The collected liquid was filtered immediately andtemporarily stored at 4°C until concentrated by rotary evaporation. The concentrated liquid was freeze-dried and extracted with methanol. After the methanol was vaporized, the obtained dry matter was dissolved again in ultra-pure water and used to determine the nitrification inhibition capacity of *L. chinensis* root exudates.

#### 2.2.2 Inhibition of *L. chinensis* root exudates on nitrification potential

The shaken soil-slurry method (Hart et al., 1994; Janke et al., 2018) was also used to determine the effects of the collected root exudates of *L. chinensis* on soil nitrification potential in *P. tenuiflora* soils, which have a high nitrification potential. The incubation system was the same as the aforementioned soil nitrification potential measurement. *P. tenuiflora* soil (3 g) and 30 mL of PN solution were filled into Erlenmeyer flasks. After the addition of different concentrations of root exudates or nitrification inhibitor, ${{\text{NO}}_{2}}^{‐}‐\text{N}$ concentration was determined using a colorimetric microplate assay (O’Sullivan et al., 2017). A relationship curve between ${{\text{NO}}_{2}}^{‐}‐\text{N}$ concentration and culture time was used to assess soil nitrification potential. Nitrification inhibition capacities were calculated as the percentage reduction in nitrification potential in each treatment relative to that in the control (O’Sullivan et al., 2017). For this experiment, there were three treatments: (1) control: treated with water instead of nitrification inhibitor; (2) RE of *L. chinensis*: treated with root exudates (RE) of *L. chinensis*; and (3) DCD: positive control, treated with dicyandiamide (DCD). Each nitrification inhibitor had three concentration levels: 1 mg mL^-1^, 0.5 mg mL^-1^, and 0.1 mg mL^-1^.

### 2.3 Rhizosphere effects of *L. chinensis* on soil nitrification

We designed a soil culture experiment of *L. chinensis* treated with NH_4_Cl. This was done considering that methanol traces can inhibit nitrification (Suzuki et al., 1976). The experiment aimed to detect the effects of *L. chinensis* root exudates on soil $\text{N}{{\text{H}}_{4}}^{+}‐\text{N}$ , ${{\text{NO}}_{3}}^{‐}‐\text{N}$ , and nitrifying microorganisms. The root exudates were allowed to directly act on microorganisms of the rhizosphere to avoid the effects of methanol.

In this experiment, a concentric rings device separated by a 500-mesh nylon net (pore size of 30 μm) was designed (the structure and usage are detailed in **Figure S2**) and used to culture *L. chinensis*. The center pillar soil was treated as the rhizosphere soil, and the soil outside of the buffer zone as the non-rhizosphere soil. The saline-alkali soils (pH 7.97 ± 0.01, $\text{N}{{\text{H}}_{4}}^{+}‐\text{N}$ 6.34 ± 0.65 mg kg^-1^, ${{\text{NO}}_{3}}^{‐}‐\text{N}$ 3.80 ± 0.31 mg kg^-1^, TOC 10.78 ± 0.42 mg g^-1^) collected from the Dongtai pasture field site were used for the soil culture experiment after being air-dried and passed through a 10-mesh sieve. For each device, the used soil mass from the center to the outside was 20 g, 40 g, and 40 g, respectively. One week after the planting of *L. chinensis*, the same concentration of NH_4_Cl solution was used to treat the soil layer once a day, with the volume from the center to the outside being 1 mL, 2 mL, and 2 mL, respectively. There were three concentrations of NH_4_Cl: 0 mM, 3 mM, and 6 mM. After the NH_4_Cl treatment, soil moisture was maintained at 80% of its saturated water content by the weighing method. Each treatment group was replicated 7 times. After 8 weeks of treatment, the concentration of $\text{N}{{\text{H}}_{4}}^{+}‐\text{N}$ and ${{\text{NO}}_{3}}^{‐}‐\text{N}$ in the rhizosphere soil and the non-rhizosphere soil was determined, respectively.

The soil $\text{N}{{\text{H}}_{4}}^{+}‐\text{N}$ concentration was determined as detailed in 2.1.2.

Since UV spectrophotometry requires rigorously calibrated quartz cuvettes and the number of samples tested is large, the soil ${{\text{NO}}_{3}}^{‐}‐\text{N}$ concentration content in this part of the experiment was determined using the high-throughput microplate technique method modified from Shand et al. (Shand et al., 2008). The extraction procedures were performed as in 2.1.2. The 50 μL supernatant was pipetted into the microplate, followed by the addition of 25 μL each of the catalyst (dissolve 35.4 mg of CuSO_4_·5H_2_O and 900 mg of ZnSO_4_·7H_2_O together in 900 mL of water and dilute to 1 L), sodium hydroxide (40 g L^-1^) and hydrazine sulphate (1.71 g L^-1^) solutions. After the mixture was incubated for 15 min at 35°C, 25 μL of *p*-aminobenzenesulfonic acid (10 g L^-1^ in 3.5 M HCl) solution and 25 μL of *α*-naphthylamine (2 g L^-1^ in 3.5 M HCl) solution were added sequentially. The optical density was measured at 520 nm, and the ${{\text{NO}}_{3}}^{‐}‐\text{N}$ concentration was determined against a range of aqueous standards (0 - 1 mg ${{\text{NO}}_{3}}^{‐}‐\text{N}$ L^-1^) prepared from KNO_3_. Soil total inorganic nitrogen (TIN) concentration was calculated as the sum of ${{\text{NO}}_{3}}^{‐}‐\text{N}$ and $\text{N}{{\text{H}}_{4}}^{+}‐\text{N}$ .

The abundance of the active nitrifying bacterial community was determined using quantification of 16S rRNA. Briefly, total soil genomic DNA was extracted using the Fast DNA^®^ SPIN Kit for Soil (MP Biomedicals, Santa Ana, CA, USA) according to the manufacturer’s instructions. The integrity of genomic DNA was detected through agarose gel electrophoresis, and the concentration and purity of genomic DNA were detected through the Nanodrop 2000 (Thermo Fisher Scientific, Massachusetts, USA) and Qubit 3.0 Spectrophotometer (Thermo Fisher Scientific, Massachusetts, USA). Multiple spike-ins with identical conserved regions to natural 16S rRNA genes and variable regions replaced by random sequence with ~40% GC contents were artificially synthesized. Then, an appropriate proportion of spike-ins mixture with known gradient copy numbers was added to the sample DNA. The V3 - V4 hypervariable regions of the 16S rRNA gene and spike-ins were amplified with the primer 515F (Illumina adapter sequence 1 + GTGCCAGCMGCCGCGG) and primer 907R (Illumina adapter sequence 2 + CCGTCAATTCMTTTRAGTTT) and then sequenced using an Illumina NovaSeq 6000 sequencer (Illumina, California, USA) (Jiang et al., 2019; Wang et al., 2020). The abundance of nitrifying bacteria (aerobic ammonia oxidation bacteria, AOB; aerobic nitrite oxidation bacteria, NOB) was predicted by FAPROTAX software (Louca et al., 2016). The raw sequencing data have been uploaded to NCBI database (BioProject ID: PRJNA930065).

### 2.4 Functions of $\text{N}{{\text{H}}_{4}}^{+}‐\text{N}$ and ${{\text{NO}}_{3}}^{‐}‐\text{N}$ in the adaptation of *L. chinensis* to alkali stress

In this study, a hydroponic system with a modified half-strength Hoagland solution was utilized. The system aimed to investigate the effects of different forms of N ( $\text{N}{{\text{H}}_{4}}^{+}‐\text{N}$ and ${{\text{NO}}_{3}}^{‐}‐\text{N}$ ) on biomass and root exudation of *L. chinensis*. Additionally, pH and inorganic N (IN) content in the culture medium under different pH conditions.

#### 2.4.1 Plant material and growth conditions

The plant materials used in this experiment were obtained from the aforementioned Dongtai pasture and transplanted into a saline-alkali soil nursery. At the beginning of the experiment, *L. chinensis* seedlings from the nursery were planted in a sandbox with a leak at the bottom and grown in a greenhouse for three months. The sandbox was thoroughly irrigated once a week in the first month and every three days in the subsequent two months. For the rest of the time, the sandbox was watered daily to maintain an appropriate moisture level. One day before pouring the nutrient solution, the sandbox was irrigated with distilled water three times the volume of the nutrient solution to wash away excess residual salt of the culture medium. A modified half-strength Hoagland solution (CaCl_2_ 1.7 mM, NaH_2_PO_4_·2H_2_O 0.5 mM, MgSO_4_ 1 mM, EDTA-FeSO_4_·7H_2_O 0.1 mM, KCl 6 mM, and mixed-N [NH_4_NO_3_ 3 mM], H_3_BO_3_ 23 μM, MnCl_2_·4H_2_O 4.6 μM, H_2_MoO_4_ 0.1 μM, ZnSO_4_·7H_2_O 0.4 μM, CuSO_4_·5H_2_O 0.2 μM, CoSO_4_·7H_2_O 0.2 μM) with pH = 8 was used. Three months later, the nutrient solutions with different N forms, including $\text{N}{{\text{H}}_{4}}^{+}‐\text{N}$ [NH_4_Cl 6 mM], ${{\text{NO}}_{3}}^{‐}‐\text{N}$ (KNO_3_ 6 mM, and KCl not added to ensure K^+^ balance), and mixed-N (NH_4_NO_3_ 3 mM) as culture medium, were prepared and divided into three parts, and their pH values were adjusted to 6, 7, and 8. *L. chinensis* seedlings at generally the same growth levels were each placed into culture tubes modified from 10-mL centrifuge tubes, with holes punched at the bottom and lids removed. After adding sand to bury the roots of the *L. chinensis* seedlings, rows of culture tubes were placed in boxes containing different nutrient solutions and cultured in a greenhouse for four weeks until the roots grew out of the holes at the bottom. The sand was then rinsed with water and the culture process continued with the same nutrient solutions for eight weeks (See **Figure S3A**). The nutrient solution was changed once a week in the first four weeks, and every 3 d in the subsequent eight weeks. Throughout the process, 20% NaOH solution was used to adjust the pH of the culture medium every day to ensure it was close to the original value. After 12 weeks of culture, samples were collected on a sunny day. On the night before sample collection, the roots of the pre-cultured *L. chinensis* with culture tube were washed with ultra-pure water three times. After excess water on the roots was dried with absorbent paper, *L. chinensis* with the culture tubes were put into 50-mL centrifuge tubes, which contained 45 mL of the corresponding nutrient solution and weighing in advance, to be pre-treated for 12 h in dark in order to adapt to the fresh nutrient solution and reduce the pulse effect of culture media replacement on the root secretion of *L. chinensis*. On the next day, the corresponding fresh nutrient solution was replaced and exposed to sunlight for 6 h (see **Figure S3B**).

#### 2.4.2 Sampling

The nutrient solution was collected after the dark and sunshine treatment, respectively. After weighing and replenishing the lost water, the pH value of the collected nutrient solution was determined by a multiparameter analyzer (DZS-706-B, Shanghai INESA Scientific Instrument Co., Ltd, Shanghai, China), and then frozen and stored for use in the determination of other indexes.

After collection of the nutrient solutions, the *L. chinensis* plants were immediately taken out and cut from the junction of the root and stem to divide them into above- and below-ground (including the fibrous roots and rhizomes) parts. After freeze-drying (FDU-2110, EYELA, Tokyo, Japan), the biomass of different parts was measured.

#### 2.4.3 Mass of root exudates

The TOC content of the collected nutrient solution was used to represent the root exudate quantity of *L. chinensis*, and the TOC contents were determined using the modified ultraviolet spectrophotometry method at 254 nm (Dobbs et al., 1972; Albrektienė et al., 2012). In brief, after thawing and centrifugation (at 9569 g) of the previously collected nutrient solution, 250 μL supernatant was pipetted into the quartz microplate, followed by the addition of 25 μL of 10% H_2_SO_4_ solution. The absorbance values were then read with a microplate reader (Synergy HT, BioTek, Winooski, VT, USA) at a wavelength of 254 nm, and the TOC concentration was determined against a range of aqueous standards (0 - 2500 mg C L^-1^) prepared from potassium hydrogen phthalate.

#### 2.4.4 Contents of $\text{N}{{\text{H}}_{4}}^{+}‐\text{N}$ , ${{\text{NO}}_{3}}^{‐}‐\text{N}$ and ${{\text{NO}}_{2}}^{‐}‐\text{N}$ in the collected nutrient solution

The $\text{N}{{\text{H}}_{4}}^{+}‐\text{N}$ content was determined as in 2.1.2 without extraction, ${{\text{NO}}_{3}}^{‐}‐\text{N}$ content was determined as in 2.1.2, and the ${{\text{NO}}_{2}}^{‐}‐\text{N}$ content was determined by the same procedure used for ${{\text{NO}}_{3}}^{‐}‐\text{N}$ as in 2.3 but omitting the hydrazine solution reduced the ${{\text{NO}}_{3}}^{‐}‐\text{N}$ . Calibration was achieved using a range of standards (0 - 1 mg N L^-1^) prepared from NaNO_2_. Residual TIN in each nutrient solution is the sum of $\text{N}{{\text{H}}_{4}}^{+}‐\text{N}$ , ${{\text{NO}}_{3}}^{‐}$ N, and ${{\text{NO}}_{2}}^{‐}‐\text{N}$ . The IN consumption per biomass of *L. chinensis* was calculated by subtracting the residual IN contents from the original IN contents of the nutrient solution, and dividing by the biomass of *L. chinensis*.

### 2.5 Statistical analysis

Statistical analyses were carried out using IBM SPSS Statistics 25 for Windows (IBM Corp., Armonk, NY, USA). Statistically significant differences among the treatments were determined using Least Significant Difference (LSD) calculations when data distribution (Shapiro-Wilk test) and homogeneity of variances (Levene’s test) assumptions were satisfied, or using Tamhane T2 multiple range tests when homogeneity of variance assumptions were not satisfied. Correlation analyses were assessed using Pearson correlation analysis. SigmaPlot 14.0 (Systat Software Inc., San Jose, CA, USA) was used for data visualization.

## 3 Results

### 3.1 Effects of *L. chinensis* on soil nitrification

With the succession proceeding, the concentrations of TIN, $\text{N}{{\text{H}}_{4}}^{+}‐\text{N}$ , ${{\text{NO}}_{3}}^{‐}‐\text{N}$ , TN, TOC, MBN, and MBC in soil had an increasing tendency. The *L. chinensis* community had the highest values in the concentrations of these nutrients except for ${{\text{NO}}_{3}}^{‐}‐\text{N}$ (**Figures 1A, B, D**). In the studied plant communities, soil ${{\text{NO}}_{3}}^{‐}‐\text{N}$ concentrations were significantly higher than those of $\text{N}{{\text{H}}_{4}}^{+}‐\text{N}$ ; although ${{\text{NO}}_{3}}^{‐}‐\text{N}$ was still the predominant form of inorganic N, the proportion of $\text{N}{{\text{H}}_{4}}^{+}‐\text{N}$ tripled in *L. chinensis* compared to the other successional stages (**Figures 1A, C**).

**Figure 2:**
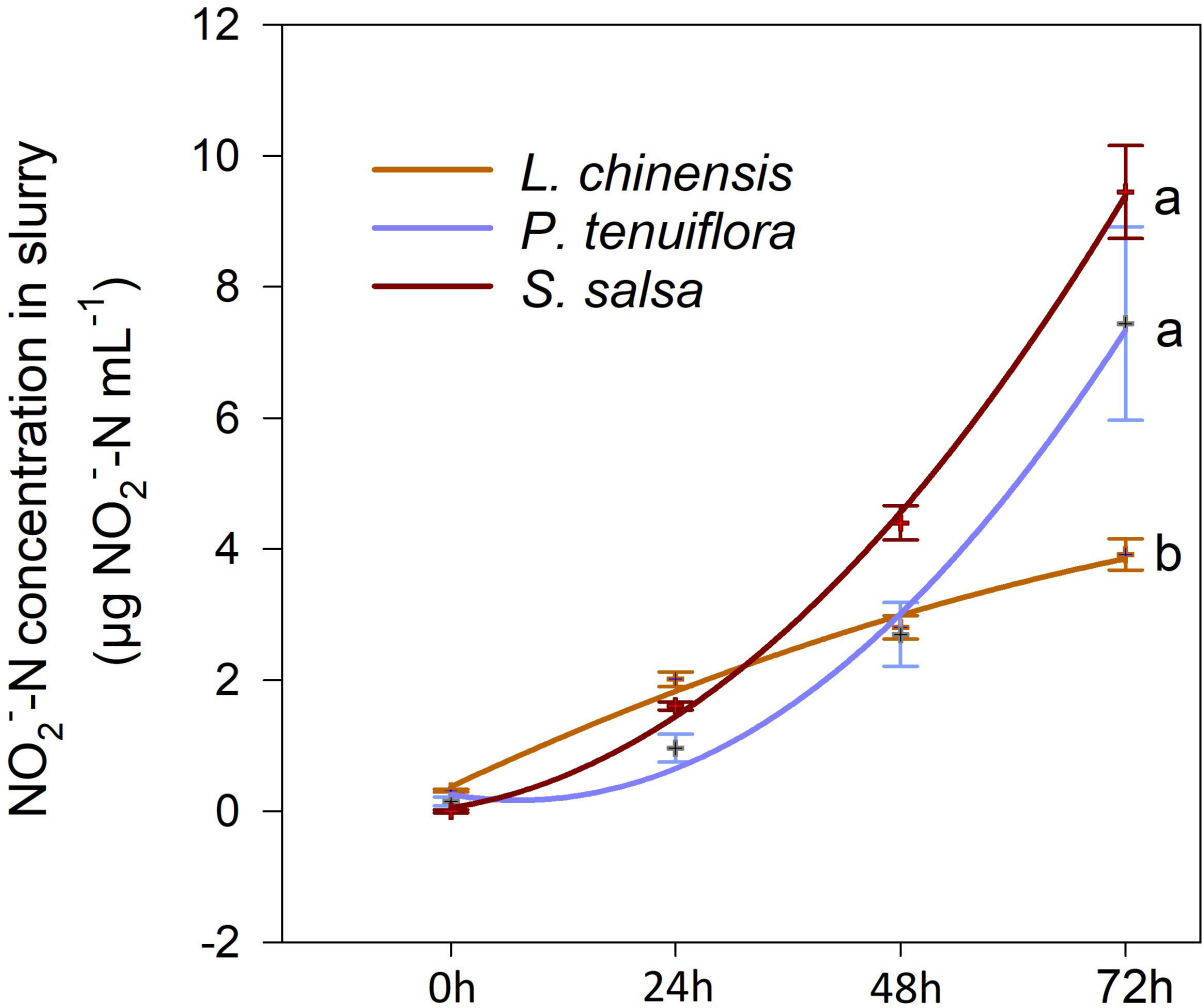
Variation of ${{\text{NO}}_{2}}^{‐}‐\text{N}$ in soil slurry with culture time in 0-10 cm soil of the three plant communities (*L. chinensis*: the final stage of vegetation succession; *P. tenuiflora:* the middle stage of succession; *S. salsa*: the initial stage of succession). Data are means ± 1 SE (n = 5). Different letters denote significant differences among the succession stages (*P* < 0.05).

There were differences in the time course variation of nitrification potential among the three plant communities, with the *L. chinensis* soil having the least increase in ${{\text{NO}}_{2}}^{‐}‐\text{N}$ concentration; however, the ${{\text{NO}}_{2}}^{‐}‐\text{N}$ concentration in the *P. tenuiflora* and *S. salsa* soils increased rapidly after 24 h of the incubation (**Figure 2**). After incubation for 72 h, the lowest soil nitrification potential was observed in the *L. chinensis* community, which was 53% that of the *P. tenuiflora* community, or 41% that of the *S. salsa* community. There were no differences in soil nitrification potential between the *P. tenuiflora* and *S. salsa* communities at 72 h.

**Figure 3:**
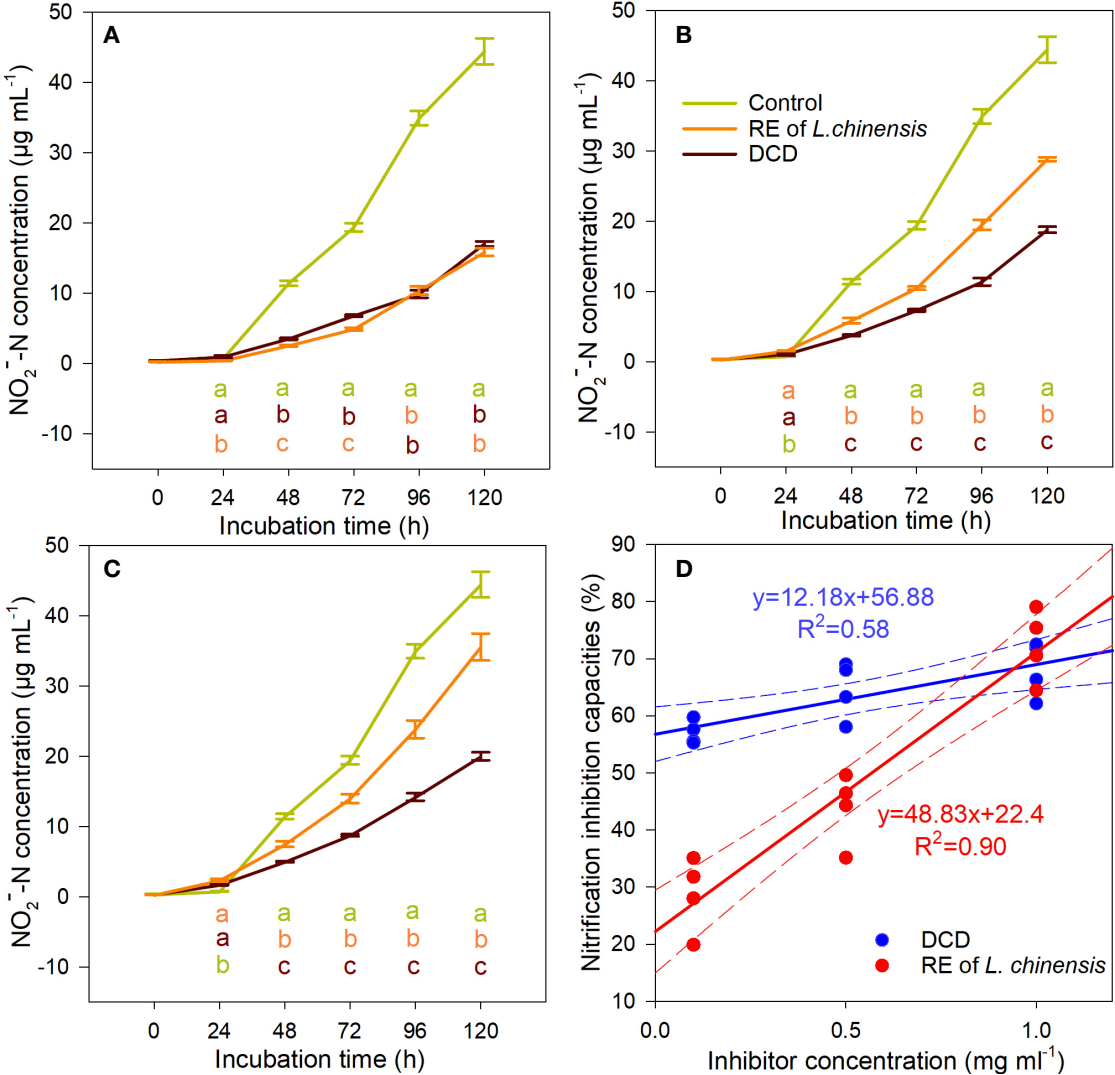
The inhibitory effect of *L. chinensis*’ root exudates at 1 mg mL^-1^ **(A)**, 0.5 mg mL^-1^ **(B)**, and 0.1 mg mL^-1^ **(C)** on the nitrification potential of soils nitrification potential of *Puccinellia tenuiflora* soil ,and the relationship between nitrification inhibitor concentration and nitrification inhibition **(D)**. Data are means ± 1 SE (n = 4). Different letters of the same color with the treatment line denote significant differences between treatments.

The presence of *L. chinensis*’ root exudates or DCD (the positive control for nitrification inhibition) inhibited the nitrification potential of the soils under *P. tenuiflora*’s influence (**Figures 3A–C**). We plotted nitrification inhibition capacities against the concentrations of DCD and root exudates of *L. chinensis*, and found that *L. chinensis* root exudates had a steeper slope (4 times greater) compared to that of the DCD, but the intercept was only 39.38% of the DCD curve (**Figure 3D**). When the concentration was close to 1 mg mL^-1^, the two curves intersected, and the average nitrification inhibition capacity of *L. chinensis* root exudates was 72.44%, higher than that of the DCD (68.29%) (**Figure 3D**). That is, for the higher *L. chinensis*’ root exudates concentration (1 mg mL^-1^), the inhibition was as efficient as that obtained with the application of DCD.

**Figure 4:**
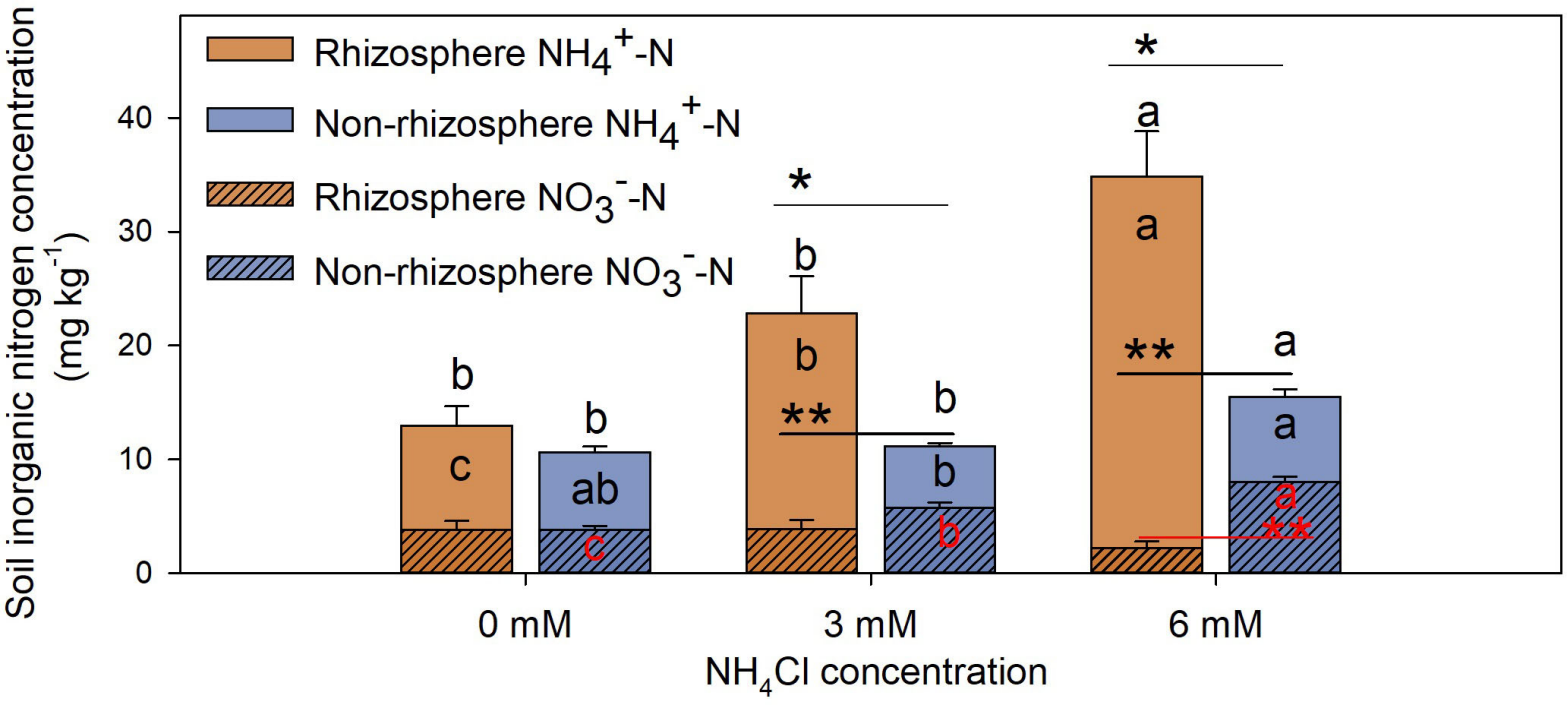
Inorganic nitrogen concentration in rhizosphere and non-rhizosphere soil of *L. chinensis* treated with different concentrations of NH_4_Cl. Data are means ± 1 SE (n = 7). Different letters denote significant differences in contents of inorganic nitrogen [black letters in the bars: $\text{N}{\text{H}}_{4}^{+}‐\text{N}$ ; red letters in the bars: ${\text{NO}}_{3}^{‐}‐\text{N}$ ; black letters above the bars: total inorganic nitrogen ( $\text{N}{{\text{H}}_{4}}^{+}‐\text{N}$ + ${{\text{NO}}_{3}}^{‐}‐\text{N}$ )] between different concentrations of ammonium nitrogen. The asterisks indicate that there are significant differences in the same N medium between rhizosphere and non-rhizosphere soil, *: *P* < 0.05; **: *P* < 0.01.

**Figure 5:**
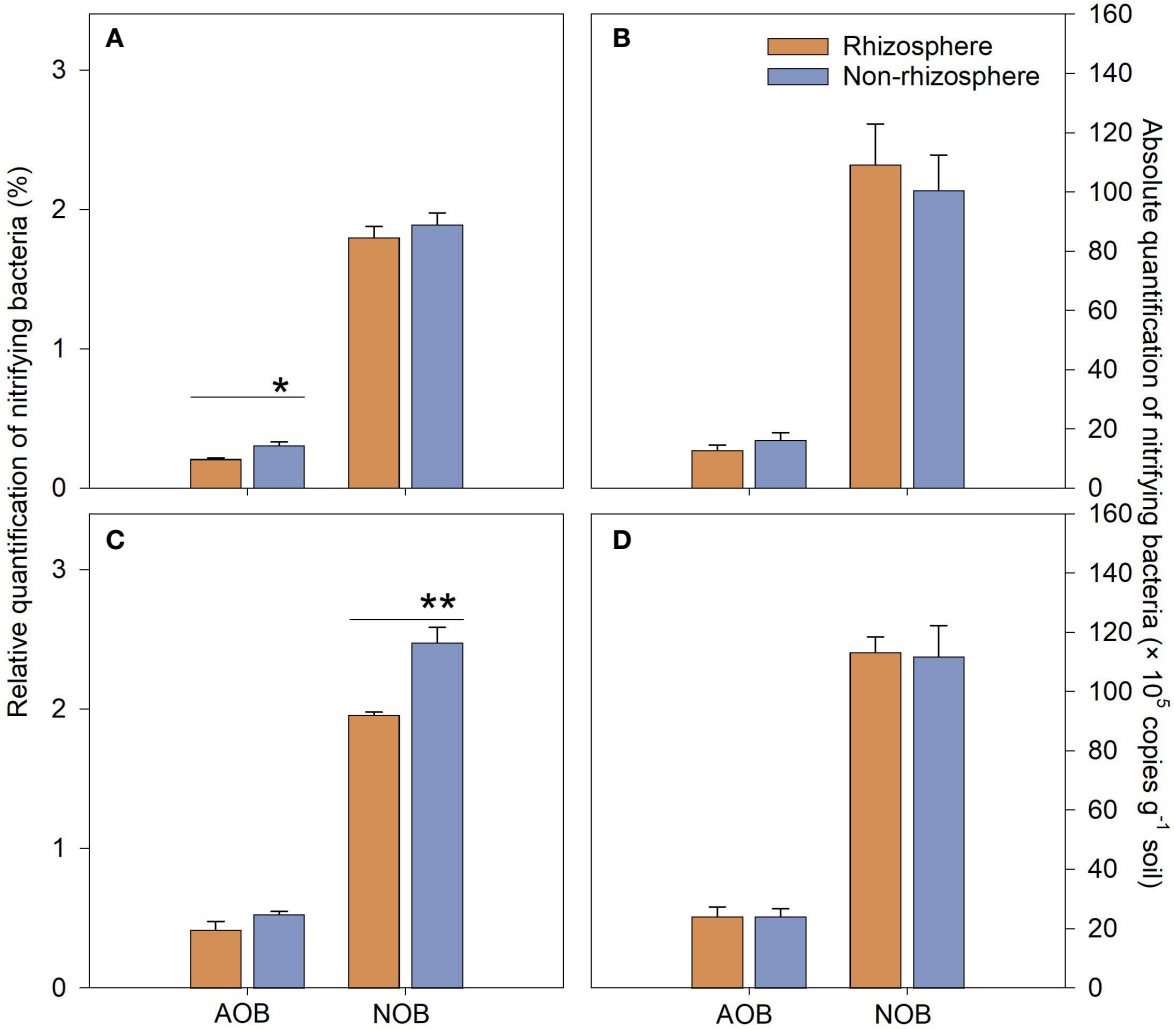
Relative **(A, C)** and absolute **(B, D)** quantification of nitrifying bacteria (AOB: ammonia oxidation bacteria, NOB: nitrite oxidation bacteria) in the rhizosphere and non-rhizosphere soil of *L. chinensis* treated with different concentrations [(3 mM: **(A, B)** 6 mM: **(C, D)**] of NH_4_Cl. Data are means ± 1 SE (n = 3). The asterisks indicate that there are significant differences between the rhizosphere and non-rhizosphere soil which treated with the same concentration of NH_4_Cl, *: *P* < 0.05; **: *P* < 0.01.

The concentration of $\text{N}{{\text{H}}_{4}}^{+}‐\text{N}$ in the rhizosphere soil of *L. chinensis* treated with different concentrations of NH_4_Cl was significantly higher than it was in the non-rhizosphere soil (**Figure 4**, *P* < 0.01). Moreover, concentrations of $\text{N}{{\text{H}}_{4}}^{+}‐\text{N}$ and TIN in the rhizosphere soil increased, whereas the concentration of ${{\text{NO}}_{3}}^{‐}‐\text{N}$ decreased with the increase in NH_4_Cl concentration (**Figure 4**, *P* < 0.05). On the contrary, the concentration of ${{\text{NO}}_{3}}^{‐}‐\text{N}$ in the non-rhizosphere soil increased significantly with the increase in NH_4_Cl concentration (**Figure 4**, *P* < 0.05). There were no significant differences in the absolute quantification (AQ) of nitrifying bacteria between the rhizosphere and non-rhizosphere soil (**Figures 5B, D**, *P* > 0.05). However, the relative quantification (RQ) of AOB in the rhizosphere soil was significantly lower than it was in the non-rhizosphere soil at the treatment of 3 mM NH_4_Cl (**Figure 5A**, *P* < 0.05), and the RQ of NOB in the rhizosphere soil was significantly lower than it was in the non-rhizosphere soil at the treatment of 6 mM NH_4_Cl (**Figure 5C**, *P* < 0.01).

### 3.2 Effects of N form on *L. chinensis* growth and adaptation to alkali stress

#### 3.2.1 Nitrate-N is more beneficial to biomass accumulation than ammonium-N

**Figure 6:**
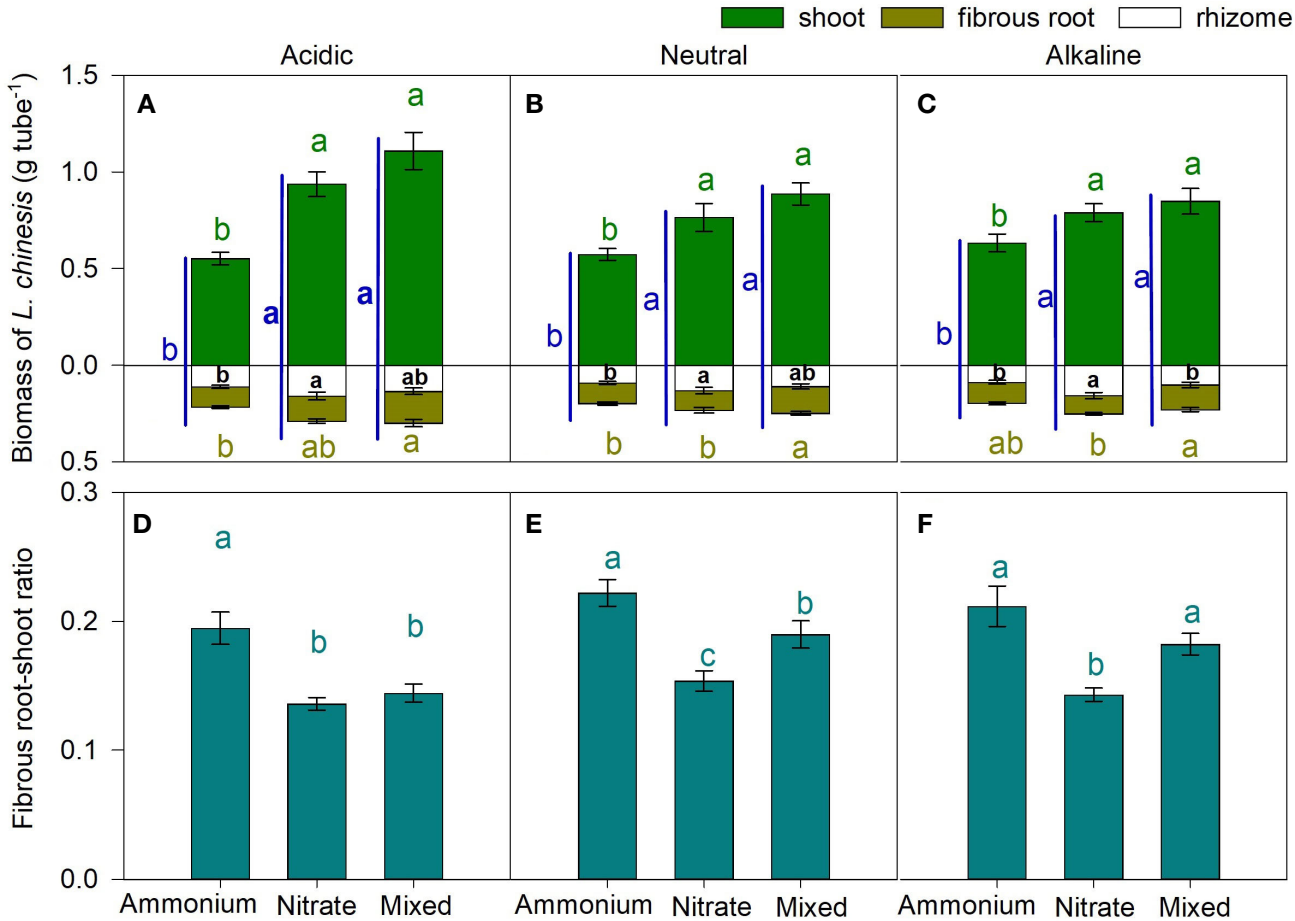
Effects of different nitrogen (N) forms (Ammonium: NH_4_Cl, Nitrate: KNO_3_, Mixed: NH_4_NO_3_) on biomass of *L. chinensis* [**(A–C)** g tube^-1^] and fibrous root-shoot ratio **(D–F)** under different pH value of the culture media [Acidic: pH = 6, **(A, D)** Neutral: pH = 7, **(B, E)** Alkaline: pH = 8, **(C, F)**]. *L. chinensis* plants were divided into three parts: shoot (green), rhizome (white), and fibrous root (brown). Data are means ± 1 SE (n = 21). Different letters of the same color above/in and beside the bars denote significant differences in weight of components or whole *L. chinensis* plant and fibrous root-shoot ratio among the N forms.

The total biomass of *L. chinensis* grown in the ${{\text{NO}}_{3}}^{‐}‐\text{N}$ containing nutrient solution was higher than that grown in $\text{N}{{\text{H}}_{4}}^{+}‐\text{N}$ , with the shoot biomass accounting for a greater proportion of the total biomass than the fibrous root or rhizome biomass (**Figures 6A–C**). The highest total biomass was detected in *L. chinensis* grown under the mixed-N; but the differences in the total biomass between the mixed-N and ${{\text{NO}}_{3}}^{‐}‐\text{N}$ were not statistically significant. With the increase in pH in the nutrient solution, the total biomass of the ${{\text{NO}}_{3}}^{‐}‐\text{N}$ -grown *L. chinensis* gradually decreased; whereas there was a trend toward increasing for the $\text{N}{{\text{H}}_{4}}^{+}‐\text{N}$ -grown *L. chinensis*.

For the studied pH conditions, the $\text{N}{{\text{H}}_{4}}^{+}‐\text{N}$ -grown plants had a higher value of the fibrous root/shoot biomass ratio compared to the ${{\text{NO}}_{3}}^{‐}‐\text{N}$ -grown *L. chinensis*. Moreover, the mixed-N treatment had greater (relative to the ${{\text{NO}}_{3}}^{‐}‐\text{N}$ treatment) ratios of fibrous root to shoot biomass when the pH of the culture medium was non-acidic (**Figures 6D–F**).

The soil of *L. chinensis* community has the highest number of total bacterial communities, which is two orders of magnitude higher than that of the *P. tenuiflora* community and three orders of magnitude higher than that of the *S. salsa* community. Similarly, the copy number of *amoA* gene in the *L. chinensis* community is still the highest, with more *amoA*-AOA than *amoA*-AOB (**Table S2**).

#### 3.2.2 Effects of ammonium-N on absorption of nitrate-N by *L. chinensis*

**Figure 7:**
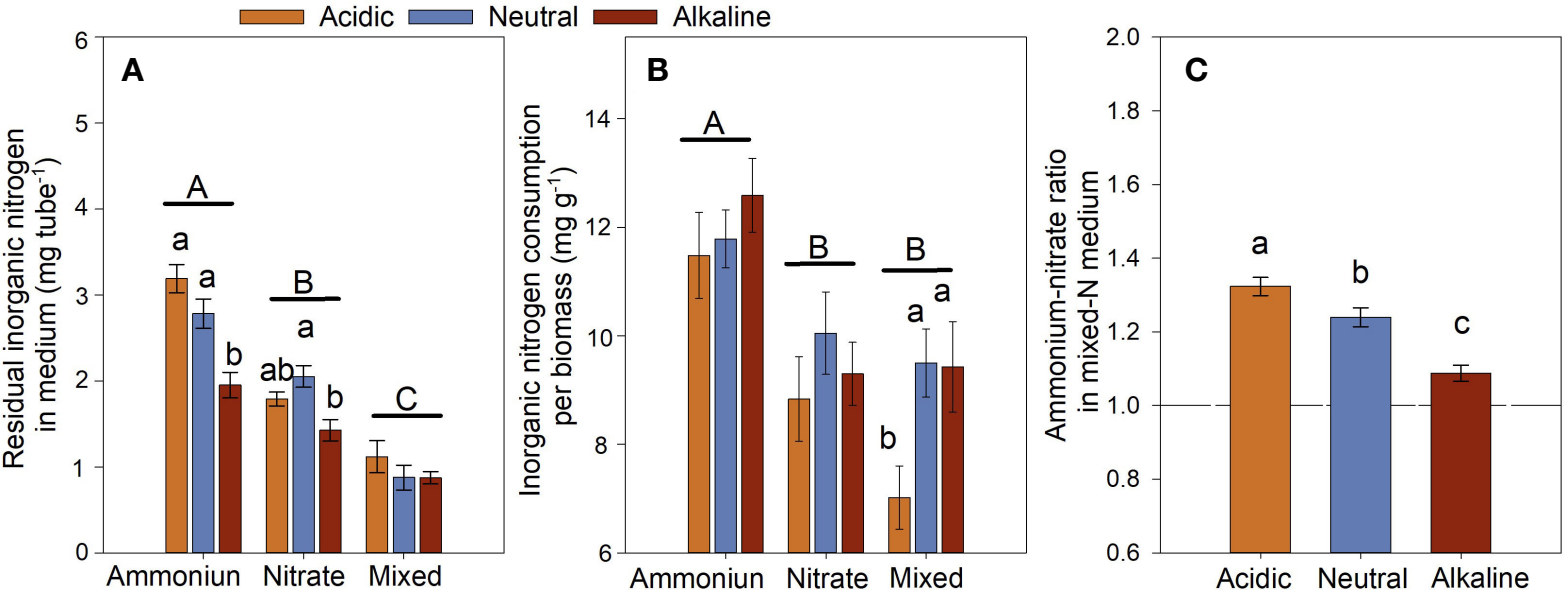
The residual inorganic nitrogen ( $\text{N}{{\text{H}}_{4}}^{+}‐\text{N}$ + ${{\text{NO}}_{3}}^{‐}‐\text{N}$ + ${{\text{NO}}_{2}}^{‐}‐\text{N}$ ) in the culture medium **(A)**, inorganic nitrogen consumption (the original inorganic-N content in the medium minus the residual inorganic-N in the medium after culture) per biomass of *L. chinensis* **(B)**, and the $\text{N}{{\text{H}}_{4}}^{+}‐\text{N}$ / ${{\text{NO}}_{3}}^{‐}‐\text{N}$ ratio in the mixed-N medium **(C)** of *L. chinensis* cultured under light for 6 h. Data are means ± 1 SE. Different small letters above the bars denote significant differences among the pH treatments in the same N form (n = 21), and different capital letters above the horizontal line indicate significant differences among the N forms (n = 63).

After culture for 6 h under the sunlight conditions, the $\text{N}{{\text{H}}_{4}}^{+}‐\text{N}$ medium had the highest value in the residual inorganic-N, whereas the mixed-N medium had the lowest value (**Figure 7A**). In other words, the total inorganic-N consumption of the $\text{N}{{\text{H}}_{4}}^{+}‐\text{N}$ -grown *L. chinensis* was significantly lower than that of the ${{\text{NO}}_{3}}^{‐}‐\text{N}$ -grown *L. chinensis*. However, inorganic-N consumption per unit biomass of the $\text{N}{{\text{H}}_{4}}^{+}‐\text{N}$ -grown *L. chinensis* was significantly higher than that of the ${{\text{NO}}_{3}}^{‐}‐\text{N}$ -grown ones; inorganic-N consumption of the mixed-N treatment was the lowest except under the alkaline conditions (**Figure 7B**). The ratio of $\text{N}{{\text{H}}_{4}}^{+}‐\text{N}$ to ${{\text{NO}}_{3}}^{‐}‐\text{N}$ in the mixed-N medium was greater than 1 under the three studied pH conditions (**Figure 7C**), indicating that *L. chinensis* absorbed more ${{\text{NO}}_{3}}^{‐}‐\text{N}$ than $\text{N}{{\text{H}}_{4}}^{+}‐\text{N}$ .

#### 3.2.3 Ammonium-N affects pH of *L. chinensis* growth environment

**Figure 8:**
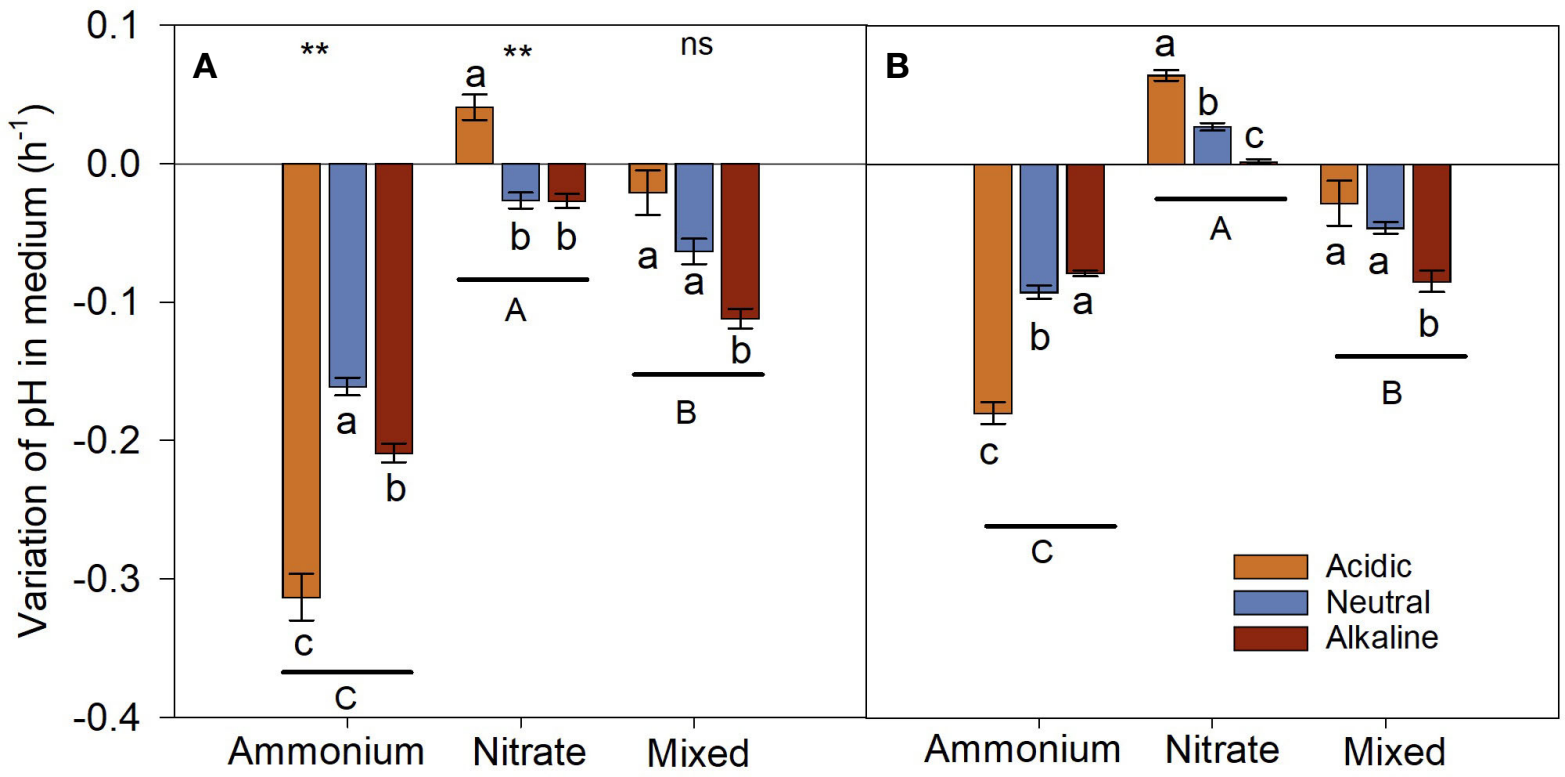
Variation in pH in the medium of *L. chinensis* cultured under the light [**(A)** Day] and dark [**(B)** Night] period. Data are means ± 1 SE. Different small letters above/below the bars denote significant differences among the pH treatments in the same N medium (n = 21), and different capital letters below the horizontal line indicate significant differences among the N medium (n = 63). The asterisks indicate that there are significant differences in the same N medium between the light and night periods, ns: *P* > 0.05; **: *P* < 0.01.

The three N treatments differed significantly in variations of culture medium pH during the sunlight and dark periods (**Figure 8**). The growth of *L. chinensis* induced a decrease in pH of culture medium treated with $\text{N}{{\text{H}}_{4}}^{+}‐\text{N}$ and mixed-N growth medium, with the magnitude of the decrease being more apparent in the $\text{N}{{\text{H}}_{4}}^{+}‐\text{N}$ medium. For the ${{\text{NO}}_{3}}^{‐}‐\text{N}$ treatment, pH value increased slightly in the acidic medium, while it decreased slightly in the neutral and alkaline media during the sunlight period; however, pH value of the acidic, neutral, and alkaline medium increased during the dark period.

#### 3.2.4 Effects of N forms on root exudate secretion

**Figure 9:**
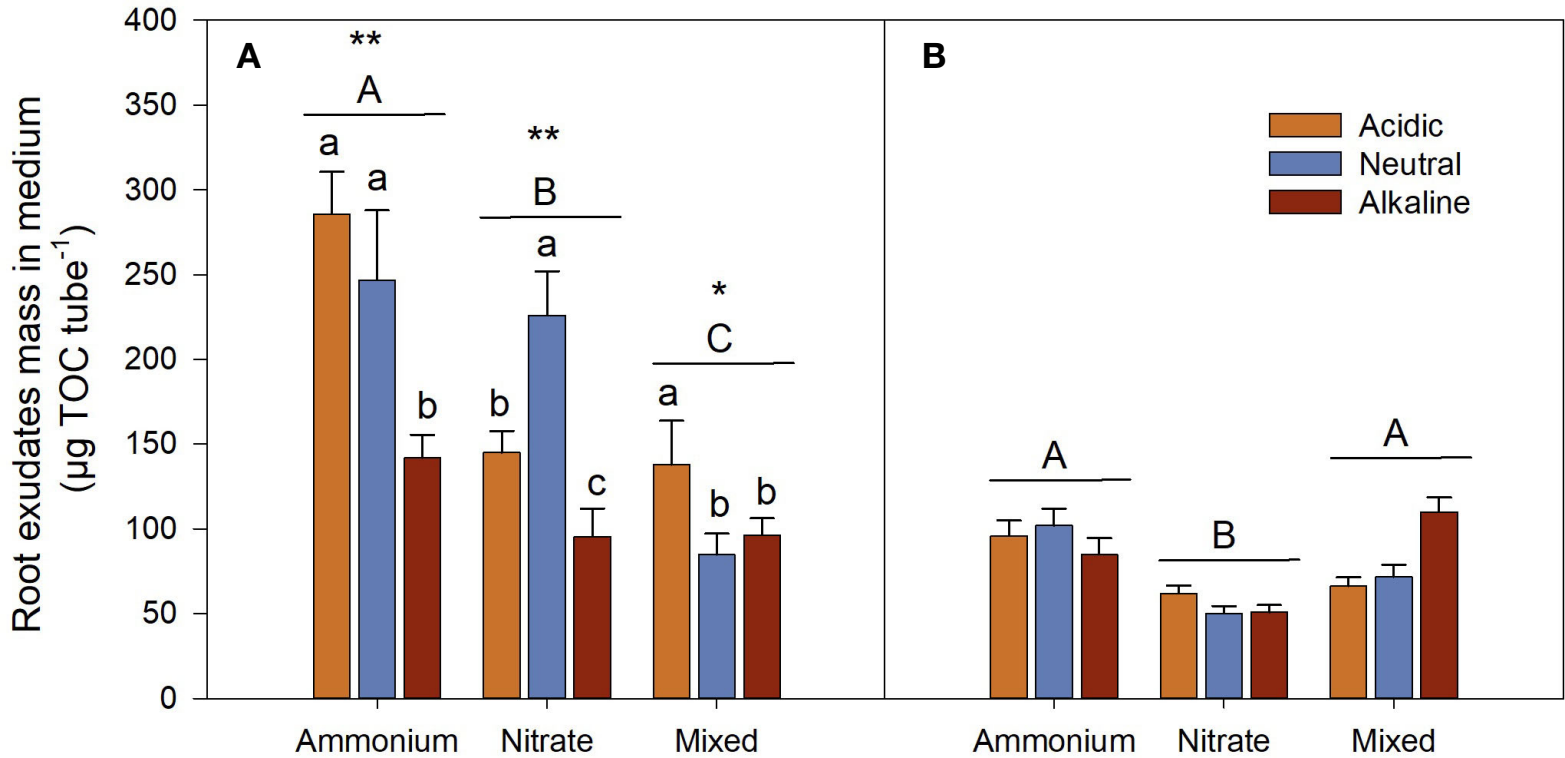
Root exudate mass in the medium of *L. chinensis* cultured under the light **(A)** Day) and dark **(B)** Night) conditions. Data are means ± 1 SE (n = 21). Different small letters denote significant differences in the same nitrogen (N) media under different pH (n = 21), and different capital letters beside the line indicate significant differences among different N media (n = 63). The asterisks indicate significant differences under the same N medium between day and night, *: *P* < 0.05; **: *P* < 0.01 (n = 63).

Root exudate mass of the $\text{N}{{\text{H}}_{4}}^{+}‐\text{N}$ -grown *L. chinensis* was significantly higher than that of the ${{\text{NO}}_{3}}^{‐}‐\text{N}$ -grown *L. chinensis*, and the mixed-N-grown *L. chinensis* had the lowest root exudate mass during the sunlight period (**Figure 9A**). Moreover, root exudate mass during the sunlight period was significantly higher than it was during the dark period for each N treatment (**Figure 9**).

## 4 Discussion

### 4.1 *L. chinensis* has BNI capacity

With the lowest soil nitrification potential (**Figure 2**), but the highest AQ of *amoA*-AOB and *amoA*-AOA (**Table S2**) and the highest concentration of $\text{N}{{\text{H}}_{4}}^{+}‐\text{N}$ (**Figure 1A**) in the soil of the *L. chinensis* community, and the presence of *L. chinensis*’ root exudates inhibited the nitrification potential of the soils under *P. tenuiflora*’s influence, which soils have a high nitrification potential (**Figure 3**), which suggest that *L. chinensis* is very likely to have BNI-capacity. However, this finding does not preclude the possibility of higher soil carbon concentration in the *L. chinensis* community (**Figures 1B, D**) with an associated increase in immobilization of $\text{N}{{\text{H}}_{4}}^{+}‐\text{N}$ and ${{\text{NO}}_{3}}^{‐}‐\text{N}$ , which resulted in a decline in net nitrification rates because of a simultaneous reduction in gross nitrification due to the decrease in nitrification substrate and the increase in ${{\text{NO}}_{3}}^{‐}‐\text{N}$ immobilization (Hart et al., 1994). Moreover, the residual trace of methanol after the extraction of root exudates may also inhibit nitrification (Suzuki et al., 1976). Thus, it seems that the BNI-capacity of *L. chinensis* remains uncertain. The results showed that the content of $\text{N}{{\text{H}}_{4}}^{+}‐\text{N}$ in the rhizosphere soil of *L. chinensis* treated with different concentrations of NH_4_Cl was significantly higher than that in the non-rhizosphere soil (**Figure 4**), and among them, the concentration of ${{\text{NO}}_{3}}^{‐}‐\text{N}$ in the non-rhizosphere soil was significantly higher than that in the rhizosphere soil when NH_4_Cl was 6 mM (**Figure 4**). Considering that microbes are preferentially immobilize $\text{N}{{\text{H}}_{4}}^{+}‐\text{N}$ than ${{\text{NO}}_{3}}^{‐}‐\text{N}$ (Jansson et al., 1955; Recous et al., 1990), and the biomass and basal respiration of microorganisms in rhizosphere is generally higher than that in non-rhizosphere (Tang et al., 2020), the immobilization of $\text{N}{{\text{H}}_{4}}^{+}‐\text{N}$ in rhizosphere should be more than that of non-rhizosphere, that is, the concentration of $\text{N}{{\text{H}}_{4}}^{+}‐\text{N}$ in rhizosphere should be lower than that in non-rhizosphere soil. However, experimentally, the opposite results have been observed. A reason for this phenomenon could be attributed to the BNI of *L. chinensis*; the higher ${{\text{NO}}_{3}}^{‐}‐\text{N}$ content in the non-rhizosphere than in the rhizosphere also supported this speculation because of the lack of inhibition of nitrification by BNIs in the non-rhizosphere soil. It was also found that there was no significant difference in the AQ of nitrifying bacteria between the rhizosphere and non-rhizosphere soil of *L. chinensis* (**Figures 5B, D**, *P* > 0.05). However, when the NH_4_Cl treatment concentration was 3 mM, the RQ of AOB in the *L. chinensis* rhizosphere soil was significantly lower than that in the non-rhizosphere soil (**Figure 5A**, *P* < 0.05), and the RQ of NOB in the rhizosphere soil was significantly lower than that in the non-rhizosphere soil when the NH_4_Cl concentration was 6 mM (**Figure 5C**, *P* < 0.01). Under the condition that there was no significant difference in nitrifying bacteria AQ between the rhizosphere and non-rhizosphere soil of *L. chinensis*, the RQ of nitrifying bacteria in the rhizosphere soil was significantly lower than that in the non-rhizosphere soil, which indicates that the total amount of bacteria in the rhizosphere soil was higher than that in the non-rhizosphere soil. The aforementioned microbes preferentially immobilize $\text{N}{{\text{H}}_{4}}^{+}‐\text{N}$ over ${{\text{NO}}_{3}}^{‐}‐\text{N}$ , and the concentration of $\text{N}{{\text{H}}_{4}}^{+}‐\text{N}$ in the rhizosphere of *L. chinensis* was still significantly higher than that in the non-rhizosphere even though *L. chinensis* takes up more N in the rhizosphere. Considering that the pH of non-rhizosphere soil (8.05 ± 0.015, n = 7) is higher than that of the rhizosphere soil (7.76 ± 0.019, n = 7), the volatilization of $\text{N}{{\text{H}}_{4}}^{+}‐\text{N}$ in the non-rhizosphere may be higher than that in the rhizosphere, but the concentration of ${{\text{NO}}_{3}}^{‐}‐\text{N}$ in the non-rhizosphere soil is significantly higher than that in the rhizosphere soil, although the ${{\text{NO}}_{3}}^{‐}‐\text{N}$ can flow to the rhizosphere, the ${{\text{NO}}_{3}}^{‐}‐\text{N}$ in non-rhizosphere soil in this study can only come from $\text{N}{{\text{H}}_{4}}^{+}‐\text{N}$ oxidized by nitrifying microorganisms. These are the results of the lack of BNIs in the non-rhizosphere soil. In addition, BNIs such as phenylalanine, α-linolenic acid, etc. were found in the root exudates of *L. chinensis* (unpublished data). Taken together, the results of our study revealed that the root exudates of *L. chinensis* has BNI-ability.

### 4.2 Adaptation of *L. chinensis* to alkali stress via BNI associated ammonium supply

As a dominant late-successional plant species in saline-alkali meadow steppe, *L. chinensis* appears to have evolved corresponding adaptation strategies for high pH and N-limited soil. The studies suggested that BNI production is a key adaptation strategy of plant root systems to limit soil-nitrifier activity and reduce ${{\text{NO}}_{3}}^{‐}‐\text{N}$ generation and associated N losses in N-limited soils (Subbarao et al., 2006b; Nardi et al., 2020; Ghatak et al., 2022). It should provide a competitive advantage for N acquisition because, firstly, positively charged NH_4_^+^ is naturally attracted to negatively charged clay surfaces, this binding is sufficiently strong to limit $\text{N}{{\text{H}}_{4}}^{+}‐\text{N}$ loss by leaching; in contrast, negatively charged ${{\text{NO}}_{3}}^{‐}$ is liable to be lost via leaching and denitrification in soil (Subbarao et al., 2012). Secondly, $\text{N}{{\text{H}}_{4}}^{+}‐\text{N}$ assimilation is energetically more efficient than ${{\text{NO}}_{3}}^{‐}‐\text{N}$ for plants, only a quarter of metabolic energy is required to metabolize one unit $\text{N}{{\text{H}}_{4}}^{+}‐\text{N}$ as compared to ${{\text{NO}}_{3}}^{‐}‐\text{N}$ (Subbarao et al., 2012). Consequently, it seems that maintaining N in $\text{N}{{\text{H}}_{4}}^{+}‐\text{N}$ form is advantageous way for plants to use N. However, NH_3_ volatilization is one of the main pathways of N loss in ecosystems; for example, > 40% of the applied N is lost as NH_3_ under certain environmental and edaphic conditions (Pan et al., 2016), a 40% increase in NH_3_ evolution with a pH change from 7.4 to 7.8, and this effect is greater for alkaline soils (Fenn and Kissel, 1973). Therefore, maintaining the $\text{N}{{\text{H}}_{4}}^{+}‐\text{N}$ form in alkaline soils is inconsistent with the original intention of plants to use the limited soil N efficiently.

We hypothesized that *L. chinensis* ^’^ BNI inhibits nitrification in soil to ensure the supply of $\text{N}{{\text{H}}_{4}}^{+}‐\text{N}$ , but *L. chinensis* does not directly use $\text{N}{{\text{H}}_{4}}^{+}‐\text{N}$ to improve NUE. Instead, it uses the H^+^ produced by the metabolism of $\text{N}{{\text{H}}_{4}}^{+}‐\text{N}$ to lower the pH of alkaline soil, and provides proton motive force to enhance the uptake of ${{\text{NO}}_{3}}^{‐}‐\text{N}$ in soil to improve NUE. Because, firstly, there are higher possibility for the volatilization of $\text{N}{{\text{H}}_{4}}^{+}‐\text{N}$ into NH_3_ in high pH soil (Mills et al., 1974), such a process also increases N loss risks. Secondly, plants that utilize $\text{N}{{\text{H}}_{4}}^{+}‐\text{N}$ theoretically use four times less energy than the plants that use ${{\text{NO}}_{3}}^{‐}‐\text{N}$ (Subbarao et al., 2012); however, such a perspective does not consider the energy consumed by plants in the synthesis and secretion of BNIs. Furthermore, the metabolic location, origin of energy consumption, and carbon skeleton requirements of assimilation of $\text{N}{{\text{H}}_{4}}^{+}‐\text{N}$ and ${{\text{NO}}_{3}}^{‐}‐\text{N}$ also should be considered. Therefore, according to our results, *L. chinensis* has BNI-capacity, and $\text{N}{{\text{H}}_{4}}^{+}‐\text{N}$ plays an important regulatory role in its adaptation to high pH, whereas the nutritional role of $\text{N}{{\text{H}}_{4}}^{+}‐\text{N}$ is far less than that of ${{\text{NO}}_{3}}^{‐}‐\text{N}$ .

#### 4.2.1 *L. chinensis* reduces rhizosphere pH of alkaline soil by assimilating ammonium-N

The uptake of $\text{N}{{\text{H}}_{4}}^{+}‐\text{N}$ helps *L. chinensis* reduce its rhizosphere pH under alkaline conditions. Some cationic nutrients have relatively low availability in high pH soils (Weil and Brady, 2017); for example, with every unit increase in pH, zinc, copper, and manganese concentrations decrease 100-fold (Fageria et al., 2002); whereas Fe concentrations decrease 1000-fold (Lindsay and Schwab, 1982). Most plant nutrients exhibit their highest availability in neutral or slightly acidic soil pH. Meanwhile, the pH of plant cells and organelles are strictly controlled by biochemistry and limited to very narrow ranges (Raven and Smith, 1976; Smith and Raven, 1979; Van Beusichem et al., 1985). Consequently, plants must neutralize or excrete excess ions from the cells for the maintenance of optimal pH in cells and organelles (Van Beusichem et al., 1988). $\text{N}{{\text{H}}_{4}}^{+}‐\text{N}$ is taken up and assimilated almost exclusively in the roots to avoid or minimize the toxic effects of $\text{N}{{\text{H}}_{4}}^{+}‐\text{N}$ (Raven and Smith, 1976; Allen and Smith, 1986). Protons are produced when $\text{N}{{\text{H}}_{4}}^{+}‐\text{N}$ is assimilated, and then H^+^ is pumped out of the root cells by H^+^-ATPases (Raven and Smith, 1976), which leads to a decrease in pH in the *L. chinensis* rhizosphere and can exactly provide an activation condition for cationic nutrients such as Fe. However, when assimilating ${{\text{NO}}_{3}}^{‐}‐\text{N}$ , plants must first reduce it to $\text{N}{{\text{H}}_{4}}^{+}‐\text{N}$ , which requires simultaneous H^+^ consumption and OH^-^ production (Van Beusichem et al., 1988). Consequently, H^+^ generated by using $\text{N}{{\text{H}}_{4}}^{+}‐\text{N}$ on one hand reduced the pH of the habitats of *L. chinensis* (**Figure 8**), on the other hand the H^+^ provides proton motive force for the uptake of ${{\text{NO}}_{3}}^{‐}‐\text{N}$ (Long et al., 2015), which is conducive to the effective use of N nutrients by *L. chinensis*. Therefore, *L. chinensis* uptakes the maximum amount of N when it is fed with mixed-N forms, compared to when it is fed with a N form alone (**Figure 7A**), and accumulates the greatest amount of biomass (**Figures 6A–C**). H^+^ generation is potentially one of the main reasons why $\text{N}{{\text{H}}_{4}}^{+}‐\text{N}$ facilitated *L. chinensis’* adaptation to high pH stress.

#### 4.2.2 Nutritional function of ammonium-N is less than nitrate-N for *L. chinensis*

It has long been recognized that N assimilation requires energy and carbon skeletons (Nunes-Nesi et al., 2010; Xu et al., 2012), and N form affects dry matter distribution and carbohydrate consumption (Guo et al., 2007). In the present study, *L. chinensis* cultured with ${{\text{NO}}_{3}}^{‐}‐\text{N}$ had significantly higher biomass than it growing with the $\text{N}{{\text{H}}_{4}}^{+}‐\text{N}$ medium (**Figure 6**). One possible cause is that a higher fraction of root carbon is used in $\text{N}{{\text{H}}_{4}}^{+}‐\text{N}$ absorption and assimilation (Bloom et al., 1992; Guo et al., 2007; Britto and Kronzucker, 2013), because plants have to detoxify $\text{N}{{\text{H}}_{4}}^{+}‐\text{N}$ in the roots by assimilating it into organic compounds via glutamine synthetase-glutamate synthetase (GS/GOGAT), and these biochemical processes create a high demand for carbon skeletons, which originate from the Tri-Carboxylic Acid (TCA) cycle in the shoot (Guo et al., 2007). Supersaturated organic compounds include amino acids (AA) synthesized by the root, and some carbohydrates synthesized by photosynthesis and transported to the roots are secreted from the root tip by passive transport (Mahmood et al., 2002). It may be one of the reasons for the observation of the highest root exudates mass of the $\text{N}{{\text{H}}_{4}}^{+}‐\text{N}$ -grown *L. chinensis* (**Figure 9A**), which leaves less reduced carbon available for *L. chinensis* growth and maintenance (Britto and Kronzucker, 2013), and leads to a relatively low biomass accumulation in the $\text{N}{{\text{H}}_{4}}^{+}‐\text{N}$ -grown *L. chinensis* (**Figures 6A–C**). This result suggests that ${{\text{NO}}_{3}}^{‐}‐\text{N}$ was more beneficial to the biomass accumulation of *L. chinensis* than $\text{N}{{\text{H}}_{4}}^{+}‐\text{N}$ . Correspondingly, the amounts of residual N in the ${{\text{NO}}_{3}}^{‐}‐\text{N}$ medium were less than those in the $\text{N}{{\text{H}}_{4}}^{+}‐\text{N}$ medium (**Figure 7A**), and there was a lower content of ${{\text{NO}}_{3}}^{‐}‐\text{N}$ compared with $\text{N}{{\text{H}}_{4}}^{+}‐\text{N}$ even if $\text{N}{{\text{H}}_{4}}^{+}‐\text{N}$ was volatilized under the alkaline condition in the mixed-N medium (**Figure 7C**). The biomass and residual N results suggest that more ${{\text{NO}}_{3}}^{‐}‐\text{N}$ than $\text{N}{{\text{H}}_{4}}^{+}‐\text{N}$ was absorbed by *L. chinensis*; and the consumption of $\text{N}{{\text{H}}_{4}}^{+}‐\text{N}$ per biomass was significantly higher than that of ${{\text{NO}}_{3}}^{‐}‐\text{N}$ (**Figure 7B**). These results provide evidence that *L. chinensis* prefers ${{\text{NO}}_{3}}^{‐}‐\text{N}$ for its growth, this is consistent with the current view that plants distributed in alkaline soil (Hawkesford et al., 2012; Zhang et al., 2018) and semi-arid habitats (Engel et al., 2019) where ${{\text{NO}}_{3}}^{‐}‐\text{N}$ is the dominant N form, prefer ${{\text{NO}}_{3}}^{‐}‐\text{N}$ , but this is inconsistent with the conclusion of a previous study that found that $\text{N}{{\text{H}}_{4}}^{+}‐\text{N}$ is preferred over ${{\text{NO}}_{3}}^{‐}‐\text{N}$ for *L. chinensis* growth (Li et al., 2018), the reasons for this discrepancy need further study. ${{\text{NO}}_{3}}^{‐}‐\text{N}$ is the main source of IN in the natural alkaline environment of this study (**Figures 1A, C**). To cope with the N limitation, *L. chinensis* has to effectively utilize the limited N. Therefore, it is reasonable for *L. chinensis* to preferentially utilize ${{\text{NO}}_{3}}^{‐}‐\text{N}$ .

However, when *L. chinensis* was cultured with ${{\text{NO}}_{3}}^{‐}‐\text{N}$ , the absorbed ${{\text{NO}}_{3}}^{‐}‐\text{N}$ was mainly transported to the shoot for assimilation, and reduction of each ${{\text{NO}}_{3}}^{‐}$ produces one OH^-^; the OH^-^ in the cytoplasm in shoots increases carboxylation and induces the synthesis of various organic acids via the TCA cycle (Van Beusichem et al., 1988). Organic acids that are transported to the roots can only be secreted from roots by active transport (Feng et al., 2020). The excretion of OH^-^ produced by ${{\text{NO}}_{3}}^{‐}‐\text{N}$ assimilation in the root alkalizes the rhizosphere and aggravates the alkali stress (**Figure 8**). Therefore, the root exudate of the ${{\text{NO}}_{3}}^{‐}‐\text{N}$ -grown *L. chinensis* was lower than that of the $\text{N}{{\text{H}}_{4}}^{+}‐\text{N}$ -grown *L. chinensis* (**Figure 9A**).

The mixed-N medium yielded the highest biomass of *L. chinensis* (**Figures 6A–C**), which is consistent with the findings of a recently published study, that is, a mixed supply of ${{\text{NO}}_{3}}^{‐}‐\text{N}$ and $\text{N}{{\text{H}}_{4}}^{+}‐\text{N}$ maximizes plant growth compared with a sole ${{\text{NO}}_{3}}^{‐}‐\text{N}$ or $\text{N}{{\text{H}}_{4}}^{+}‐\text{N}$ supply (Wang et al., 2019). It has been suggested that, in addition to increasing photosynthesis, mixed N supply enhances shoot growth via increased auxin synthesis to build a large sink for carbon and nitrogen utilization, which, in turn, facilitates further carbon assimilation and nitrogen uptake (Wang et al., 2019). Ruan et al. (2016) found that the net uptake of ${{\text{NO}}_{3}}^{‐}‐\text{N}$ was facilitated by the co-provision of $\text{N}{{\text{H}}_{4}}^{+}‐\text{N}$ compared with ${{\text{NO}}_{3}}^{‐}‐\text{N}$ alone in tea (*Camellia sinensis*) roots. However, Kronzucker et al. (1999) found that ${{\text{NO}}_{3}}^{‐}‐\text{N}$ promoted the net uptake of $\text{N}{{\text{H}}_{4}}^{+}‐\text{N}$ compared with $\text{N}{{\text{H}}_{4}}^{+}‐\text{N}$ alone in rice, and net ${{\text{NO}}_{3}}^{‐}‐\text{N}$ uptake was significantly inhibited by the co-provision of $\text{N}{{\text{H}}_{4}}^{+}‐\text{N}$ in barley and rice, as compared to that observed with ${{\text{NO}}_{3}}^{‐}‐\text{N}$ alone at a pH of 6.5. In our opinion, the uptake of ${{\text{NO}}_{3}}^{‐}‐\text{N}$ can also be facilitated by $\text{N}{{\text{H}}_{4}}^{+}‐\text{N}$ for *L. chinensis*, because the uptake of $\text{N}{{\text{H}}_{4}}^{+}‐\text{N}$ produces protons and helps maintain the proton motive force that is required for ${{\text{NO}}_{3}}^{‐}‐\text{N}$ absorption (Hawkesford et al., 2012). However, further experiments are required to address this possibility. The mixed-N medium had the lowest amounts of residual N after the culturation of *L. chinensis* (**Figure 7A**), suggesting that the mixture of $\text{N}{{\text{H}}_{4}}^{+}‐\text{N}$ and ${{\text{NO}}_{3}}^{‐}‐\text{N}$ was more beneficial to the uptake of *L. chinensis* compared with supplying ${{\text{NO}}_{3}}^{‐}‐\text{N}$ alone. It is likely that the interactions between ${{\text{NO}}_{3}}^{‐}‐\text{N}$ and $\text{N}{{\text{H}}_{4}}^{+}‐\text{N}$ in a mixed-N medium optimize the N utilization of *L. chinensis*. Of course, this result does not exclude some NH_3_ volatilization loss under alkaline conditions, this might also be a reason why the N consumption per biomass of *L. chinensis* in the non-acid medium is significantly higher than that in the acid medium (**Figure 7B**).

**Figure 10:**
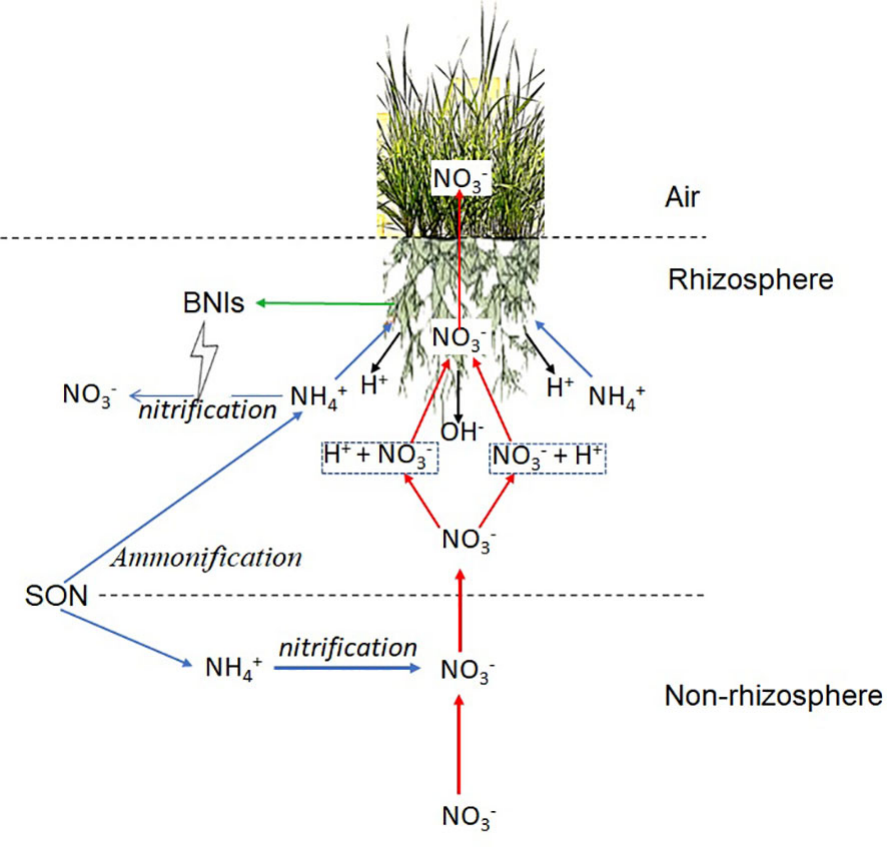
A model of pH regulation and nitrate-N ( ${{\text{NO}}_{3}}^{‐}‐\text{N}$ ) uptake by ammonium-N ( $\text{N}{{\text{H}}_{4}}^{+}‐\text{N}$ ) assimilation based on the biological nitrification inhibition (BNI)-capacity of *L. chinensis*. $\text{N}{{\text{H}}_{4}}^{+}‐\text{N}$ transport and assimilation pathways are indicated in blue arrows, nitrate-N ( ${{\text{NO}}_{3}}^{‐}‐\text{N}$ ) transport and assimilation are indicated in red arrows, and H^+^ or OH^-^ production are indicated in black arrows, BNIs secretion is indicated by green arrow. Ammonification and nitrification in non-rhizosphere soil actively took place, while nitrification in rhizosphere soil was inhibited by BNIs secreted by *L. chinensis*. $\text{N}{{\text{H}}_{4}}^{+}‐\text{N}$ is assimilated mainly in roots, which produces H^+^, which deduces the pH of rhizosphere and provides proton motive force for the uptake of ${{\text{NO}}_{3}}^{‐}‐\text{N}$ . ${{\text{NO}}_{3}}^{‐}‐\text{N}$ is taken up mainly in shoots, and most of the ${{\text{NO}}_{3}}^{‐}‐\text{N}$ absorbed by roots with the proton motive force is directly transported to shoot for uptake, some of the ${{\text{NO}}_{3}}^{‐}‐\text{N}$ was taken up in root, OH^-^ was produced then excrete into rhizosphere of *L. chinensis*. SON, soil organic nitrogen; BNIs, biological nitrification inhibitors.

Altogether, for *L. chinensis*, the $\text{N}{{\text{H}}_{4}}^{+}‐\text{N}$ utilization system is relatively low in efficiency. $\text{N}{{\text{H}}_{4}}^{+}‐\text{N}$ needs to be transported from the soil to the roots to synthesize AA. Subsequently, the AA, mainly glutamine, are loaded again and transported to shoots to participate in the fixation of CO_2_ and synthesize organic compounds. Finally, a higher fraction of the organic compounds are transported to the roots as energy and carbon skeletons for $\text{N}{{\text{H}}_{4}}^{+}‐\text{N}$ assimilation. Supersaturated organic compounds in roots, mainly AA and sugars, are secreted from the root tip by passive transport without energy-consuming, leaving less reduced carbon available for shoot growth and maintenance. In contrast, the ${{\text{NO}}_{3}}^{‐}‐\text{N}$ utilization system in *L. chinensis* is more efficient because ${{\text{NO}}_{3}}^{‐}‐\text{N}$ is taken up and transported rapidly to shoots, where it has sufficient energy and carbon skeleton supply. The organic compounds generated by ${{\text{NO}}_{3}}^{‐}‐\text{N}$ supply are mainly organic acids, which can only be secreted from roots by active transport with energy-consuming (Feng et al., 2020), so the root exudate of the ${{\text{NO}}_{3}}^{‐}‐\text{N}$ -grown *L. chinensis* was lower than that of the $\text{N}{{\text{H}}_{4}}^{+}‐\text{N}$ -grown (**Figure 9A**). As a result, more biomass of ${{\text{NO}}_{3}}^{‐}‐\text{N}$ -grown *L. chinensis* was accumulated. However, ${{\text{NO}}_{3}}^{‐}‐\text{N}$ can also be assimilated in roots and may aggravate the alkalization of the *L. chinensis* rhizosphere, which is not conducive to the uptake and utilization of other nutrient elements. Conversely, $\text{N}{{\text{H}}_{4}}^{+}‐\text{N}$ uptake by *L. chinensis* can produce large amounts of H^+^, which reduces the rhizosphere pH of *L. chinensis* under alkaline conditions and provides proton motive force for the absorption of essential nutrients, such as ${{\text{NO}}_{3}}^{‐}$ , SO_4_^2-^ (Long et al., 2015), and PO_4_^3-^ (Delhaize et al., 2015; Johri et al., 2015).If $\text{N}{{\text{H}}_{4}}^{+}‐\text{N}$ and ${{\text{NO}}_{3}}^{‐}‐\text{N}$ exist simultaneously, plants can exploit the available nutrients based on their strengths and weaknesses. In fact, there is no “exclusive ammonium environment” in nature (Marino and Fernando, 2019) due to the ubiquity of nitrifying microorganisms, especially in saline-alkali environments, where nitrification is intense and ${{\text{NO}}_{3}}^{‐}‐\text{N}$ accounts for a large proportion of inorganic-N (Kyveryga et al., 2004; Sahrawat, 2008). To adapt to the high pH stress and mineral nutrient deficiency in alkaline environments, *L. chinensis* has evolved a physiological adaptation strategy: inhibition of nitrification in the rhizosphere by secretion of BNIs from roots to enhance $\text{N}{{\text{H}}_{4}}^{+}‐\text{N}$ supply, followed by $\text{N}{{\text{H}}_{4}}^{+}‐\text{N}$ metabolism to regulate pH and the uptake of other nutrients (**Figure 10**).

## 5 Conclusions

Our results suggest that *L. chinensis* has BNI capacity. *L. chinensis* secretes BNIs not only to meet its nutritional demands for $\text{N}{{\text{H}}_{4}}^{+}‐\text{N}$ , but also because $\text{N}{{\text{H}}_{4}}^{+}‐\text{N}$ facilitates its adaptation to an alkaline environment. In association with $\text{N}{{\text{H}}_{4}}^{+}‐\text{N}$ uptake, *L. chinensis* generates and releases H^+^, which can reduce rhizosphere pH, activate cationic nutrients, and provide proton motive force for plants to ${{\text{NO}}_{3}}^{‐}‐\text{N}$ , thus improving the utilization efficiency of ${{\text{NO}}_{3}}^{‐}‐\text{N}$ . *L. chinensis* uses BNIs to ensure $\text{N}{{\text{H}}_{4}}^{+}‐\text{N}$ supply and assimilating, and maintaining proton motive force that is essential for ${{\text{NO}}_{3}}^{‐}‐\text{N}$ absorption. Therefore, the key benefit of the secretion of BNIs by *L. chinensis* is that $\text{N}{{\text{H}}_{4}}^{+}‐\text{N}$ uptake is used to regulate growth and adaptation to the alkali environment, whereas ${{\text{NO}}_{3}}^{‐}‐\text{N}$ utilization fulfills nutritional requirements.

## Data availability statement

The original contributions presented in the study are publicly available. This data can be found here: https://www.ncbi.nlm.nih.gov/sra/PRJNA930065.

## Author contributions

GW and WS designed the experiment. GW, LZ, ZG, DS, HZ, YY and SX collected data. GW analyzed data. GW and WS wrote the manuscript. JM, TY, HC, JL and ZG commented on the manuscript. All authors contributed to the article and approved the submitted version.
