# Supplementary material for: Benefits of biological nitrification inhibition of Leymus chinensis under alkaline stress: the regulatory function of ammonium-N exceeds its nutritional function
Source: Front Plant Sci. 2023 May 15;14:1145830. doi: 10.3389/fpls.2023.1145830 (PMC10225694; doi:10.3389/fpls.2023.1145830)
Supplement: Supplementary file 1 [file DataSheet_1.docx]

**
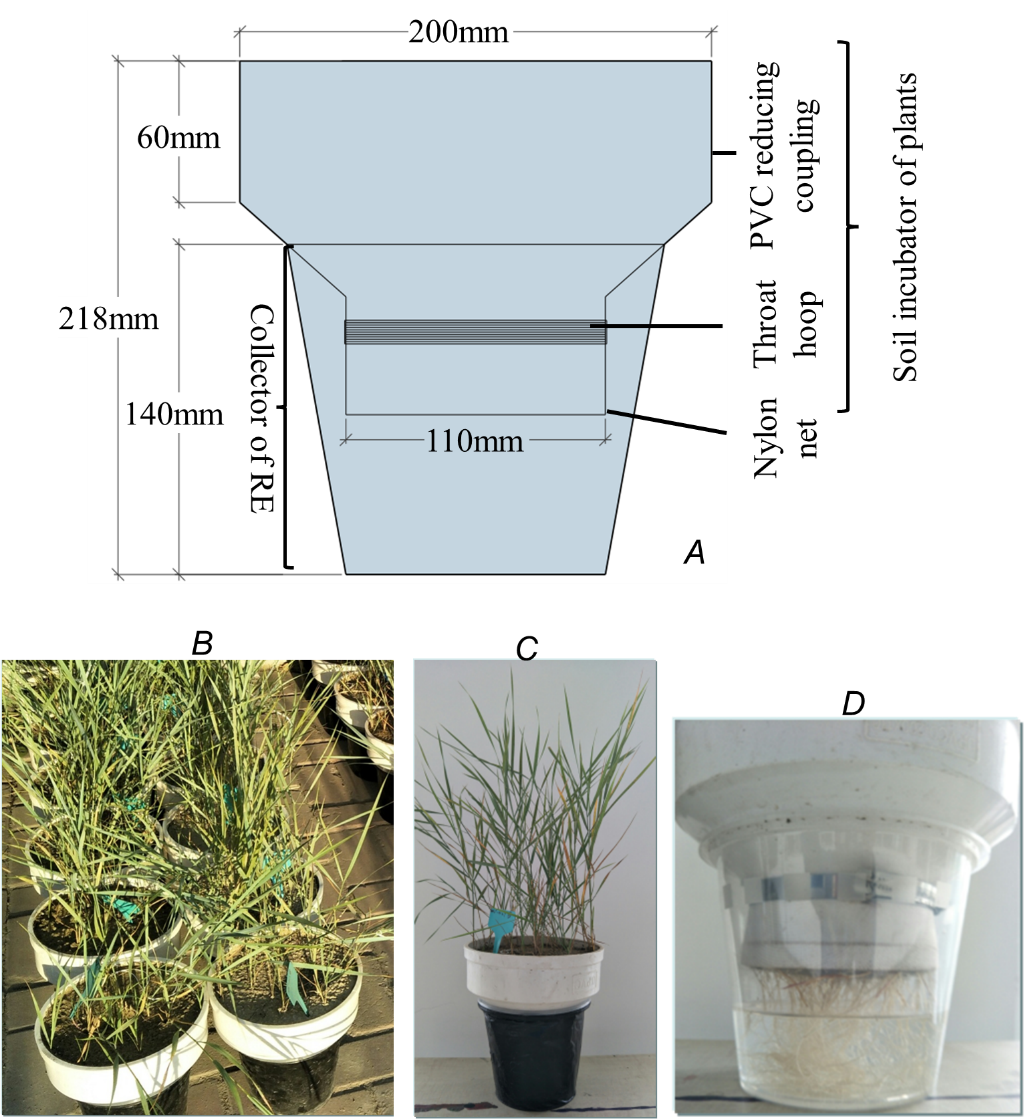
**

**Figure S1.** A diagram of plant culture device for root exudate (RE) collection (*A*) and pictures of culture devices growing with *Leymus chinensis* and RE collection (*B*, *C* and *D*). The device is composed by an upper and a lower part (*A*). The upper part is a soil incubator of plants, whose main body is a PVC reducing coupling, and the small extremity was covered by nylon mesh which has an aperture of 1 mm and was fixed with a 304-stainless steel throat hoop net. It was filled with soil to culture plants. The lower part is a RE collector made by a disposable bucket (PP) with an untransparent outer cover. The collector was filled with water and almost touched the upper nylon mesh.

**Figure S2.** The longitudinal profile diagram of the device for soil culture of *L. chinensis* separated by concentric rings (*A*) and the process of its manufacture and utilization (*B*). The roots of *L. chinensis* were confined by the center tube nylon net to grow in the center soil pillar, the water and nutrients could normally pass through the nylon net. Outside of the center soil pillar was a circle of soil separated with nylon net was used as a buffer zone, and the soil outside the buffer zone was least affected by *L. chinensis* roots.

**F**
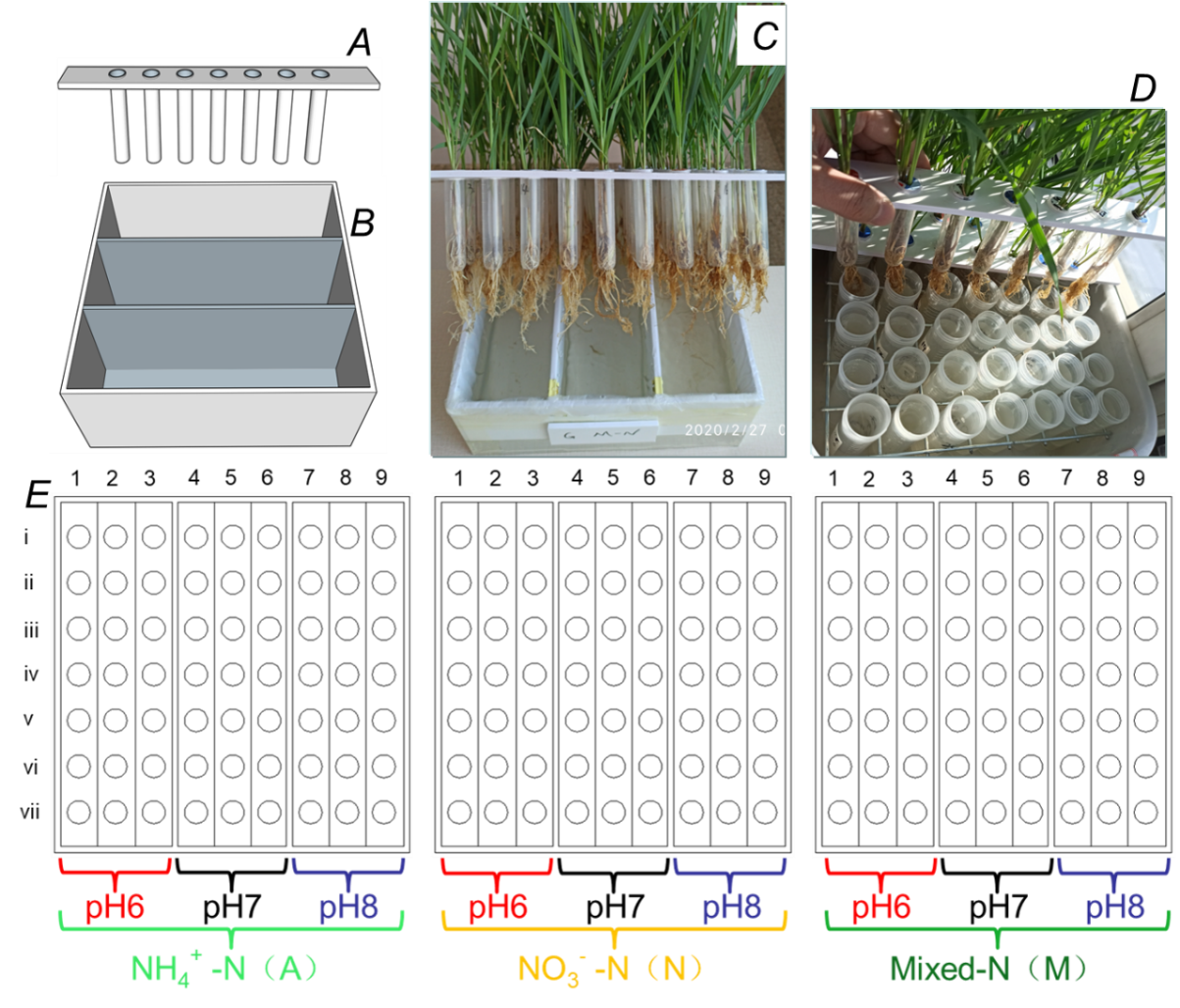
**igure S3.** Diagrams of the hydroponic device (*A* & *B*) and pictures of *Leymus chinensis* growing in the hydroponic device (*C* & *D*). An illustration of the nitrogen and pH treatments (*E*). The culture tube was a modified 10-mL centrifuge tube punched at the bottom with the lid removed, and then fixed in holes of KT rows, with seven tubes per row (*A*). After planting of the *L. chinensis* seedlings, rows of culture tubes were placed in a box made with KT plate.

**Table S1.** Vegetation features at different degradation levels in the degraded Horqin grassland.

| Dominant  species of  each vegetation | Soil  pH | Soil  EC  (μs cm^−1^) | Aboveground  biomass  (g m^−2^) | Belowground  biomass  (g m^−2^) | Companion  species |
| --- | --- | --- | --- | --- | --- |
| *L. chinensis* | 8.43 ± 0.05 c | 390.4 ± 1.21 c | 469.91 ± 20.62 a | 708.95 ± 92.66 a | *Calamagrostis epigeios* |
| *P. tenuiflora* | 9.58 ± 0.01 b | 796.2 ± 9.52 b | 193.78 ± 20.87 b | 413.48 ± 93.20 b | *Suaeda glauca*, *Chloris virgata* |
| *S. salsa* | 10.34 ± 0.01 a | 1128.6 ± 7.69 a | 163.69 ± 18.01 c | 60.11 ± 9.26 c | *Phragmites australis, Chloris virgata* |

Note: Different small letters denote significant differences between different succession stages (*P* < 0.05). Data show means ± standard errors (n = 8).

**Table S2.** Copy numbers of 16S rRNA, *amoA*-AOB and *amoA*-AOA genes in soil of different succession stages.

| succession vegetation | 16S rRNA | *amoA*-AOB | *amoA*-AOA |
| --- | --- | --- | --- |
| *L. chinensis* | 2.55×10^9^ ± 2.22×10^8^ a | 1.02×10^5^ ± 1.65×10^4^ a | 4.73×10^8^ ± 1.17×10^8^ a |
| *P. tenuiflora* | 8.48×10^7^ ± 2.46×10^7^ b | 1.26×10^4^ ± 3.92×10^3^ b | 8.15×10^5^ ± 2.96×10^5^ b |
| *S. salsa* | 3.90×10^6^ ± 1.43×10^6^ c | 3.91×10^3^ ± 1.12×10^3^ b | 7.04×10^4^ ± 2.55×10^4^ b |

Note: Different small letters denote significant differences between different succession stages (*P* < 0.05). Values are means ± 1 standard error (SE) (n = 10).
